# Neutral and Cationic Complexes of Silicon(IV) Halides with Phosphine Ligands

**DOI:** 10.1021/acs.inorgchem.2c02949

**Published:** 2022-10-12

**Authors:** Rhys P. King, John M. Dyke, William Levason, Gillian Reid

**Affiliations:** School of Chemistry, University of Southampton, Southampton SO17 1BJ, UK

## Abstract

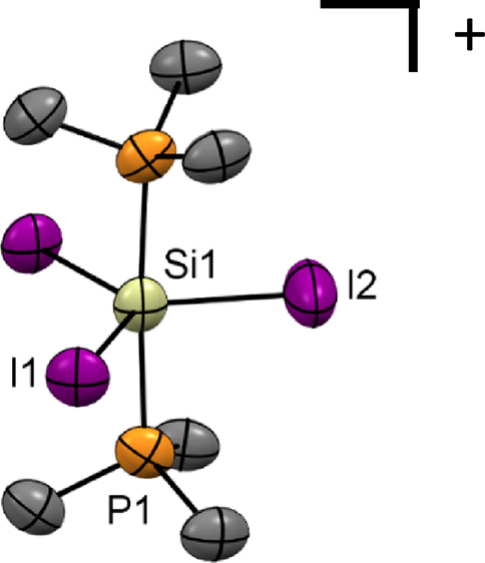

The reaction of SiI_4_ and PMe_3_ in *n*-hexane produced the yellow salt, [SiI_3_(PMe_3_)_2_]I, confirmed from its X-ray structure, containing a trigonal bipyramidal cation with *trans*-phosphines. This contrasts with the six-coordination found in (the known) *trans*-[SiX_4_(PMe_3_)_2_] (X = Cl, Br) complexes. The diphosphines *o*-C_6_H_4_(PMe_2_)_2_ and Et_2_P(CH_2_)_2_PEt_2_ form six-coordinate *cis*-[SiI_4_(diphosphine)], which were also characterized by X-ray crystallography, multinuclear NMR, and IR spectroscopy. Reaction of *trans*-[SiX_4_(PMe_3_)_2_] (X = Cl, Br) with Na[BAr^F^] (BAr^F^ = [B{3,5-(CF_3_)_2_C_6_H_3_}_4_]) produced five-coordinate [SiX_3_(PMe_3_)_2_][BAr^F^], but while Me_3_SiO_3_SCF_3_ also abstracted chloride from *trans*-[SiCl_4_(PMe_3_)_2_], the reaction products were six-coordinate complexes [SiCl_3_(PMe_3_)_2_(OTf)] and [SiCl_2_(PMe_3_)_2_(OTf)_2_] with the triflate coordinated. X-ray crystal structures were obtained for [SiCl_3_(PMe_3_)_2_][BAr^F^] and [SiCl_2_(PMe_3_)_2_(OTf)_2_]. The charge distribution across the silicon species was also examined by natural bond orbital (NBO) analyses of the computed density functional theory (DFT) wavefunctions. For the [SiX_4_(PMe_3_)_2_] and [SiX_3_(PMe_3_)_2_]^+^ complexes, the positive charge on Si decreases and the negative charge on X decreases going from X = F to X = I. Upon going from [SiX_4_(PMe_3_)_2_] to [SiX_3_(PMe_3_)_2_]^+^, i.e., removal of X^–^, there is an increase in positive charge on Si and a decrease in negative charge on the X centers (except for the case X = F). The positive charge on P shows a slight decrease.

## Abstract 2

Unusual neutral and monocationic silicon(IV) iodide complexes with soft phosphine coordination are formed in hydrocarbon solvents, while treatment of [SiCl_4_(PMe_3_)_2_] with NaBAr^F^ forms the [SiCl_3_(PMe_3_)_2_]^+^ cation; the electronic structures of [SiX_4_(PMe_3_)_2_] and [SiX_3_(PMe_3_)_2_]^+^ (X = F, Cl, Br, I) are probed using DFT calculations.

## Introduction

In marked contrast to its lighter congener (carbon), silicon forms many compounds in which the silicon center is five- or six-coordinate. These hypervalent silicon(IV) compounds, that is, compounds in which the silicon center formally exceeds eight electrons in its outer shell, have remained a very active research area for over a century and range from organosilicon species^1^ to inorganic silicon anions such as [SiF_5_]^−^, [SiF_6_]^2–^,^2^ and [Si(OTf)_6_]^2–^ (OTf^–^ = CF_3_SO_3_^–^),^3^ to complexes with bi- or poly-dentate anionic N- or O-donor ligands.^4^ Silicon(IV) halides form many hypervalent adducts with Lewis bases, including both neutral and cationic species, the majority containing neutral N- (amine, N-heterocycles, etc.) or O-donor (ethers, pnictogen oxides, etc.) ligands.^2^ Significant recent attention has focused on N-heterocyclic carbene (NHC) adducts of SiX_4_ (X = F, Cl, or Br), which, in addition to their high stability, can be reduced under appropriate conditions to generate rare examples of stable solid Si(II) compounds and even in some examples giving formally Si(I) or Si(0) species.^5^ Heterocyclic silylenes (Si(II) species) have also been described.^5^

Although only one example of displacement of fluoride from SiF_4_ by a neutral ligand is known, in [SiF_3_(Me_3_-tacn)][SiF_5_] (Me_3_-tacn = 1,4,7-trimethyl-1,4,7-triazacyclononane),^6^ silicon(IV) cations containing the heavier halogens have been known for over 50 years, typical examples being [SiCl_2_(2,2′-bipy)_2_]Cl_2_, [Si(2,2′-bipy)_3_]I_4_, [SiI_2_(py)_4_]I_2_, and [SiCl_2_(L)_2_]Cl_2_ (L = *N*-methylimidazole).^2^ More recent studies have reported complexes such as *mer*- and *fac*-[SiCl_3_(hmpa)_3_]^+^ and [SiCl_3_(hmpa)_2_]^+^ (hmpa = hexamethylphosphoramide),^7^ [SiCl_3_(Me_3_-tacn)][OTf] (OTf = CF_3_SO_3_), and [SiX_3_(pmdta)][BAr^F^] (X = Cl, Br; pmdta = Me_2_N(CH_2_)_2_N(Me)(CH_2_)_2_NMe_2_; BAr^F^ = [B{3,5-(CF_3_)_2_C_6_H_3_}_4_]).^8^ The reaction of the carbene, 1,3-dimethylimidazolidin-2-ylidene (NHC_1_), with SiCl_4_ forms the 1:1 adduct, [SiCl_4_(NHC_1_)], which reacts with the electron-deficient silane, H_2_Si(C_2_F_5_)_2_, to generate the trigonal bipyramidal cation in [SiCl_2_H(NHC_1_)_2_][SiCl_3_(C_2_F_5_)_2_], and with BCl_3_ forms [SiCl_3_(NHC_1_)][BCl_4_], containing a tetracoordinate cation. The latter reacts with further [SiCl_4_(NHC_1_)] to give the trigonal bipyramidal [SiCl_3_(NHC_1_)_2_][BCl_4_].^9^ 1,2-Bis(2,6-di-isopropylphenyl)imidazole-2-ylidene, (NHC_2_), displaces one of the halides from the silicon center to form [SiX_3_(NHC_2_)][X] (X = Br, I), which can be reduced with KC_8_ to form the neutral Si(II) species [SiX_2_(NHC_2_)]. [SiI_2_(NHC_2_)] can be reacted further with NHC_3_ (NHC_3_ = 1,3,4,5-tetramethyl-imidazol-2-ylidene) to form [Si(NHC_3_)_3_][I]_2_ featuring a very unusual Si(II) dication.^10^

Surprisingly, little work on phosphine complexes of silicon halides has been reported (and there are no known arsine adducts).^11^ Early work produced [SiX_4_(PMe_3_)_2_] (X = Cl or Br), by reaction of the constituents at low temperatures, which were identified by vibrational spectroscopy as *trans* isomers and confirmed by a low-precision X-ray crystal structure of the chloride.^12^ No complexation occurred between SiF_4_ and PMe_3_ at ambient temperatures, but tensiometric and Raman studies suggested both 1:1 and 1:2 adducts formed at low temperature (198 K), although neither was obtained pure.^12^ The preparation of [SiCl_3_(PMe_3_)_2_][ClO_4_] was also reported, but with minimal characterization.^12^ No further studies of these complexes were reported until our investigation^13^ of the reaction of SiF_4_ with a range of phosphine and diphosphine ligands, which found no evidence for adduct formation at ambient temperatures in the absence of a solvent, or in solution down to 180 K. However, detailed characterizations of *trans*-[SiX_4_(PMe_3_)_2_] (X = Cl, Br) were reported, although attempts to isolate the corresponding complexes of SiI_4_ from CH_2_Cl_2_ solution were unsuccessful.^14^

Diphosphines *o*-C_6_H_4_(PMe_2_)_2_ and R_2_P(CH_2_)_2_PR_2_ (R = Me, Et) also reacted with SiCl_4_ and SiBr_4_ to form *cis*-[SiX_4_(diphosphine)] as stable complexes, while the reaction of SiCl_4_ with the methylene-linked Me_2_PCH_2_PMe_2_ formed *trans*-[SiCl_4_(κ^1^-Me_2_PCH_2_PMe_2_)_2_], irrespective of the reactant ratio used. The first example of a phosphine complex of a halosilane, [SiHCl_3_{Et_2_P(CH_2_)_2_PEt_2_}], has also been characterized, although PMe_3_ and other diphosphines caused disproportionation into the SiCl_4_ adducts.^14^ Despite considerable efforts, the reaction of [SiCl_4_(PR_3_)_2_] or *cis*-[SiX_4_(diphosphine)] with reducing agents including KC_8_, K, sodium naphthalide, and [(DiPPNacNac)Mg]_2_ (DiPPNacNac = Ar*NC(*Me*)CHC(Me)NAr*; Ar* = 2,6-^i^Pr_2_-C_6_H_3_) did not lead to the isolation of Si(II) phosphine adducts.^14^

We report here the preparation and spectroscopic and structural characterization of several neutral and cationic complexes of silicon(IV) iodide with phosphine ligands, together with synthesis of the corresponding cations derived from chloride abstraction from silicon(IV) chloride phosphine species. Density functional theory (DFT) calculations with natural bond orbital (NBO) analysis are used to probe their electronic structures, relative energies of the frontier orbitals and the charge distributions.

## Results and Discussion

### Silicon Iodide Complexes

**Figure 1:**
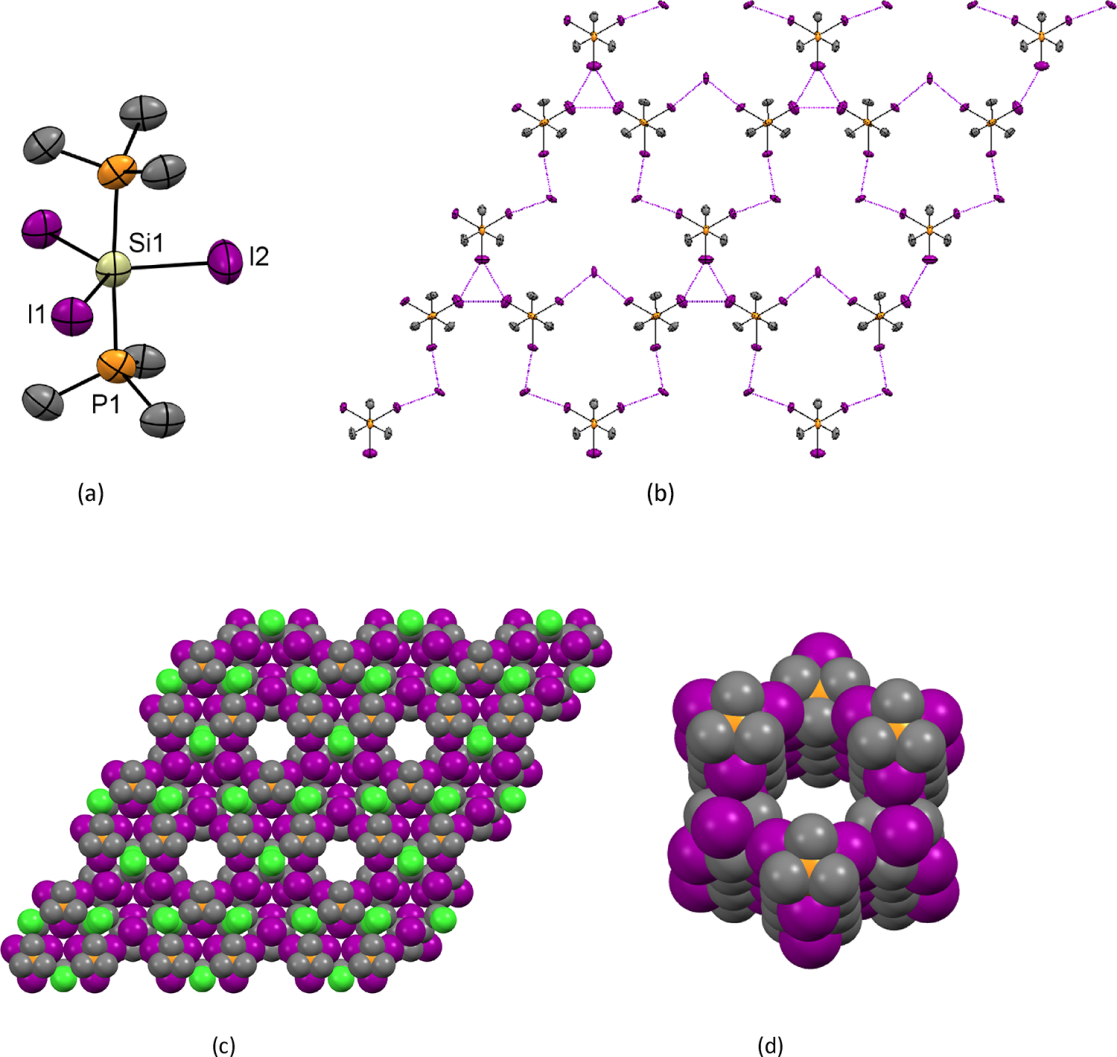
(a) Structure of the molecular cation in [SiI_3_(Me_3_P)_2_][I]·CH_2_Cl_2_·0.5C_6_H_14_ showing the atom numbering scheme. H atoms and lattice solvent molecules are omitted for clarity. Ellipsoids are drawn at the 50% probability level. Selected bond lengths (Å) and angles (°): Si1–I1 = 2.503(4), Si1–I2 = 2.471(7), Si1–P1 = 2.380(5), I1–Si1–I1 = 120.2(3), I2–Si1–I1 = 119.92(14), P1–Si1–I1 = 90.92(10), P1–Si1–I2 = 88.2(2), P1–Si1–P1 = 176.3(4); (b) view down the *c*-axis showing the weak I···I interactions (purple dashes) between the [SiI_3_(PMe_3_)_2_]^+^ cations, as well as between the [SiI_3_(PMe_3_)_2_]^+^ cations and I^–^; (c) space-filling diagram (including the CH_2_Cl_2_ solvate) showing the channels viewed down the *c*-axis. Color key: orange = P, purple = I; gray = C, green = Cl; yellow = Si; (d) view down one of the channels showing the interior lining.

**Scheme 1:**
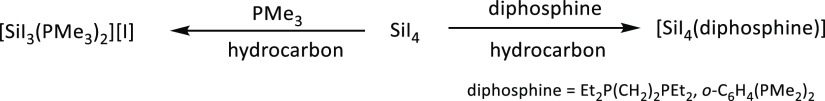
Reaction of SiI_4_ with Monodentate and Bidentate Phosphines

As noted above, although *trans*-[SiX_4_(PMe_3_)_2_] (X = Cl, Br) are readily made from SiX_4_ and PMe_3_ in anhydrous CH_2_Cl_2_, similar reactions using SiI_4_ did not yield the analogous iodide complex.^14^ However, mixing solutions of SiI_4_ and PMe_3_ in anhydrous *n*-hexane or *n*-pentane (Scheme 1) produced an immediate yellow precipitate of empirical formula SiI_4_(PMe_3_)_2_. Yellow, very moisture sensitive crystals were obtained from a CH_2_Cl_2_ solution layered with *n*-hexane and, in contrast to the known six-coordinate *trans*-[SiX_4_(PMe_3_)_2_],^14^ were found to contain a trigonal bipyramidal cation with axial phosphines with an iodide counteranion, i.e., [SiI_3_(PMe_3_)_2_]I (Figure 1). The crystallographically equivalent Si–P bonds are slightly longer than the bond lengths in the tetrahalide complexes [SiX_4_(PMe_3_)_2_] (X = Cl, Br),^14^ which probably reflects the weaker Lewis acidity of silicon iodide compared to the lighter halides, despite the positive charge in the monocation.

Within the crystal lattice, there are several long, weak intermolecular I···I interactions that fall just within the sum of the van der Waals radii (4.08 Å).^15^ These form a 2D network, as shown in Figure 1b, in which three [SiI_3_(PMe_3_)_2_]^+^ cations form weakly associated triangles *via* I···I contacts between the coordinated iodides, the shortest I···I distances being 3.933(3) Å. In addition, there are cation–anion interactions between two of the iodine atoms in each [SiI_3_(PMe_3_)_2_]^+^ unit, with two iodide counter anions, with d(I···I) = 3.6852(15) Å. These interactions lead to the formation of an extended structure containing both enclosed void cavities and two types of 1D channel, both of which are aligned down the *c* direction. Collectively, these account for ca. 39% of the cell volume. One set of 1D channels is filled with the CH_2_Cl_2_ solvent; these channels occur where the edges of the hexagonal units meet (see Figure 1c,d). The second type of 1D channel occurs at the center of the hexagonal unit. The enclosed void cavities contain electron density accounted for by applying a solvent mask and are attributed to the disordered *n*-hexane solvent (which is also present in the ^1^H NMR spectrum).

A strong peak in the far IR spectrum at 379 cm^–1^ is assigned as the *E*′ Si–I stretching vibration of the trigonal bipyramidal cation. The ^1^H NMR spectrum shows a doublet at δ = 1.66, a significant high-frequency coordination shift of +0.77 ppm, while the ^31^P{^1^H} NMR spectrum exhibits a broad singlet at δ = 3.2, a coordination shift of +65 ppm, although no silicon satellites were seen at either 293 or 180 K; ^29^Si satellites were observed in the ^31^P{^1^H} NMR spectrum recorded at 253 K (^1^*J*_SiP_ = 93 Hz). However, no convincing silicon-29 resonance could be observed over the temperature range 293–180 K, possibly due to a dynamic exchange process.

**Figure 2:**
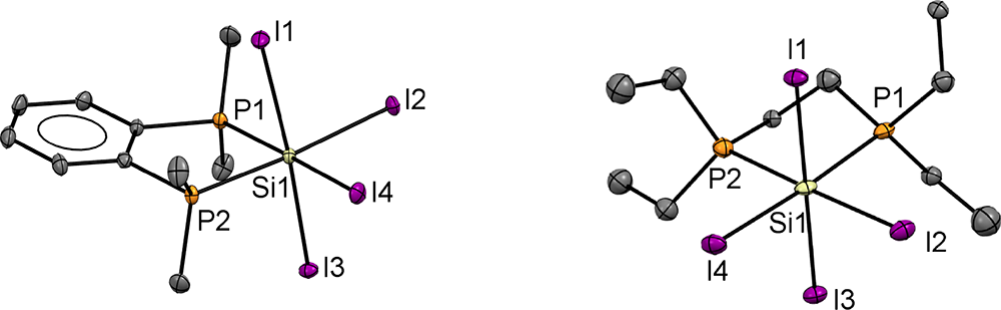
The molecular structures of (a) [SiI_4_{*o*-C_6_H_4_(PMe_2_)_2_}]·0.5C_6_H_14_ and (b) [SiI_4_{Et_2_P(CH_2_)_2_PEt_2_}] showing the atom labeling schemes. Ellipsoids are drawn at the 50% probability level, and H atoms and hexane solvent are omitted for clarity. Selected bond lengths (Å) and angles (°) for (a) are Si1–P1 = 2.3707(9), Si1–P2 = 2.3678(9), Si1–l1 = 2.6812(7), Si1–l2 = 2.6326(7), Si1–l3 = 2.6267(7), Si1–l4 = 2.6371(7), P1–Si1–P2 = 86.14(3), I1–Si1–I3 = 175.81(3), and I2–Si1–I4 = 93.47(2) and (b) are Si1–P1 = 2.391(8), Si1–P2 = 2.405(7), Si1–l1 = 2.647(7), Si1–l2 = 2.631(5), Si1–l3 = 2.670(7), Si1–l4 = 2.645(6) P1–Si1–P2 = 86.6(3), I1–Si1–3 = 177.2(2), and I2–Si1–I4 = 93.29(18).

The addition of either of the diphosphines, Et_2_P(CH_2_)_2_PEt_2_ and *o*-C_6_H_4_(PMe_2_)_2_, to a solution of SiI_4_ in a hydrocarbon solvent led to the immediate precipitation of orange solids in good yields, which were identified as [SiI_4_{Et_2_P(CH_2_)_2_PEt_2_}] and [SiI_4_{*o*-C_6_H_4_(PMe_2_)_2_}], respectively, on the basis of crystal structure determinations and microanalysis. Crystals of [SiI_4_(*o*-C_6_H_4_{PMe_2_}_2_)] were grown by layering a CH_2_Cl_2_ solution with *n*-hexane, while crystals of [SiI_4_{Et_2_P(CH_2_)_2_PEt_2_}] were obtained from slow evaporation of a CH_2_Cl_2_ solution of the complex. The structures (Figure 2) show that like their analogs with the lighter halides,^14^ they contain *cis*-octahedral molecules.

**Figure 3:**
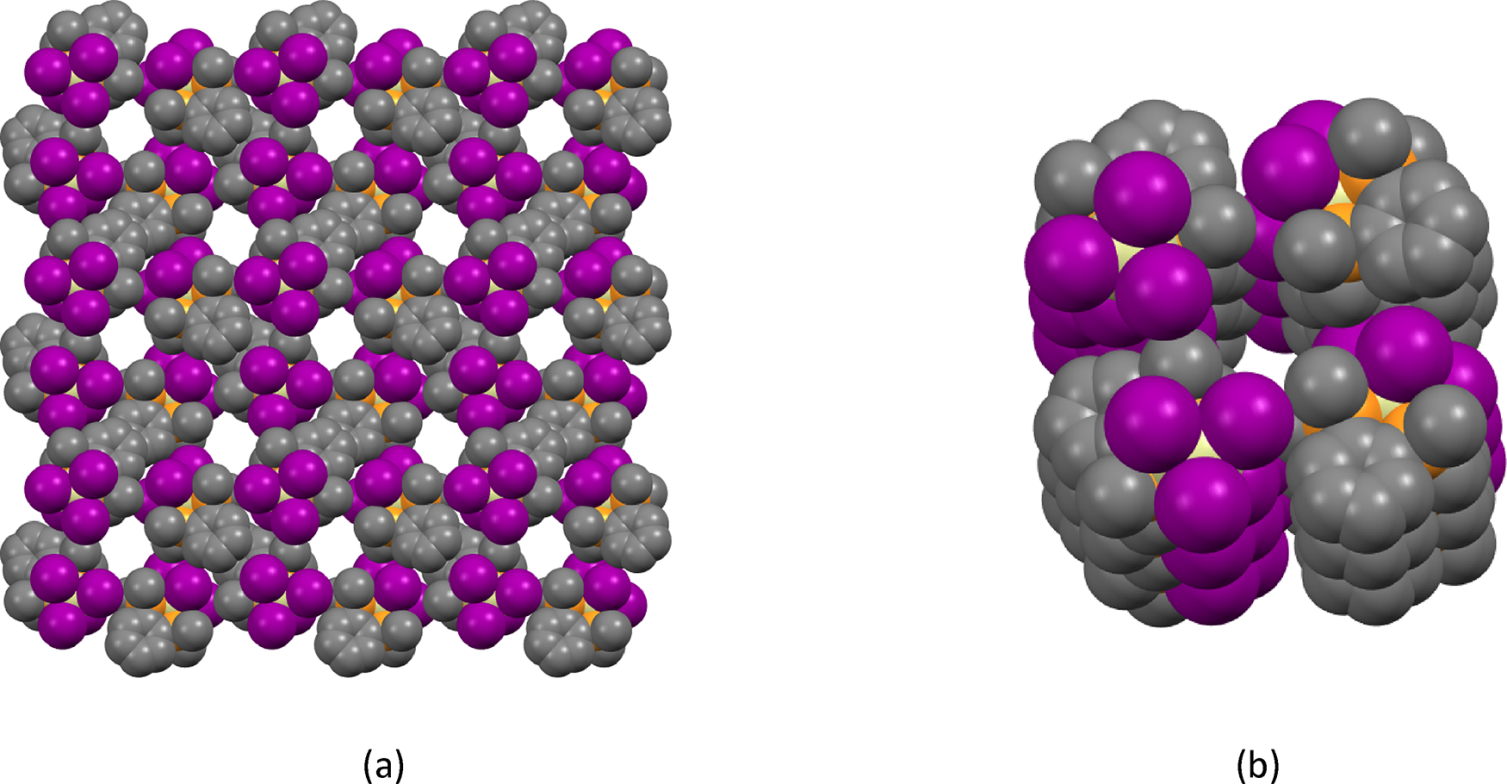
(a) Space-filling diagram of [SiI_4_{*o*-C_6_H_4_(PMe_2_)_2_}]·0.5C_6_H_14_ showing the channels viewed down the *a*-axis. Color key: orange = P; purple = I; gray = C; yellow = Si; (b) view down one of the channels showing the interior lining.

The Si–I bond distances are similar in the two [SiI_4_(diphosphine)] complexes, and all fall within the range of 2.6266(7)–2.6812(7) Å, i.e., elongated relative to SiI_4_ itself (2.43 Å),^16^ consistent with the higher coordination number present. The d(Si–I) values in the bidentate phosphine complexes are also ca. 0.15 Å longer than in the five-coordinate [SiI_3_(PMe_3_)_2_]^+^ cation described above (2.471(7)–2.503(4) Å) and can also be explained by the lower coordination number (less steric crowding) in the PMe_3_ complex, along with the presence of the positive charge strengthening the Si–I interaction. The d(Si–P) values are very similar to those in the corresponding complexes of SiX_4_ (X = Cl, Br).^14^ For [SiI_4_{*o*-C_6_H_4_(PMe_2_)_2_}] the crystal packing gives rise to a structure that contains 1D channels aligned in the *a*-direction (Figure 3). The electron density in these channels is consistent with there being two hexane molecules per unit cell.

The ^31^P{^1^H} NMR data are shown in Table 1 along with data reported for the lighter halide analogs. Notably, the iodide complexes did not exhibit convincing ^29^Si NMR resonances over the temperature range 295–183 K, possibly due to dissociative anion exchange.

**Table 1:** ^31^P{^1^H} NMR data^a^

complex	X = Cl δ(^31^P/ppm), (^1^J_PSi_/Hz)^b^	X = Br δ(^31^P/ppm), (^1^J_PSi_/Hz)^b^	X = I δ(^31^P/ppm), (^1^J_PSi_/Hz)
[SiX_4_{*o*-C_6_H_4_(PMe_2_)_2_}]	–11.9, (138)	–14.9, (103)	–33.6, (not resolved)
[SiI_4_{Et_2_P(CH_2_)_2_PEt_2_}]	+0.7, (134)	–2.1, (99)	–25.6, (not resolved)

a CD_2_Cl_2_ solution, 298 K.

b Ref (14)

### Phosphine Complexes of Silicon(IV) Cations

**Figure 4:**
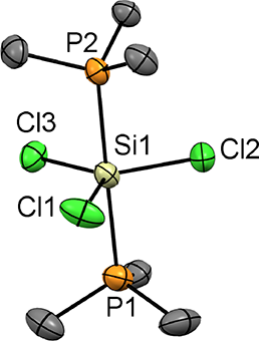
The molecular structure of the cation in [SiCl_3_(PMe_3_)_2_][BAr^F^] showing the atom labeling scheme. Ellipsoids are drawn at the 50% probability level. H atoms and the BAr^F^ counter anion are omitted for clarity. Selected bond lengths (Å) and angles (°) are Si1–P1 = 2.3272(16), Si1–P2 = 2.3229(15), Si1–Cl1 = 2.0828(15), Si1–Cl2 = 2.0811(15), Si1–Cl3 = 2.0866(14), P1–Si1–P2 = 178.97(7), Cl1–Si1–Cl2 = 120.30(7), Cl2–Si1–Cl3 = 121.27(7), and Cl1–Si1–Cl3 = 118.42(7).

**Scheme 2:**
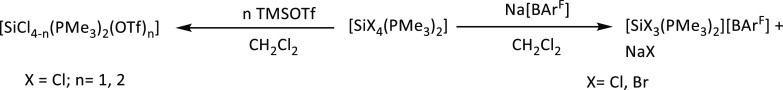
Reactions of [SiX_4_(PMe_3_)_2_] with Halide Abstraction Agents

Since phosphine-substituted cations of silicon(IV) chloride or bromide do not form spontaneously, the use of halide abstraction reagents, AlX_3_, TMSOTf (Me_3_SiO_3_SCF_3_), and Na[BAr^F^], which have been used successfully to generate tin(IV) and germanium(IV) cations,^17^ was explored. The reactions of AlX_3_ with the neutral silicon phosphine complexes caused loss of phosphine to the aluminum and were not pursued. However, addition of solid Na[BAr^F^] to a solution of [SiX_4_(PMe_3_)_2_] (X = Cl, Br) in anhydrous CH_2_Cl_2_ resulted in a white precipitate (NaX) (Scheme 2) and workup of the filtrate gave [SiX_3_(PMe_3_)_2_][BAr^F^]. Crystals of [SiCl_3_(PMe_3_)_2_][BAr^F^] were grown from a CH_2_Cl_2_ solution layered with *n*-hexane, and single crystal X-ray structure analysis showed it to have a trigonal bipyramidal cation (Figure 4).

There is a small decrease in the Si–P bond length upon going from the neutral tetrachloride complex to the monocation (Table 2). This is accompanied by a substantial decrease in the Si–Cl bond distance by ca. 0.14 Å upon formation of the monocation, indicating that most of the positive charge is taken up by the SiCl_3_ unit.

**Table 2:** Selected Geometric Parameters for Silicon Halide PMe_3_ Complexes

	[SiCl_4_(PMe_3_)_2_]^14^	[SiCl_3_(PMe_3_)_2_][BAr^F^]	[SiI_3_(PMe_3_)_2_][I]
d(Si–X), Å	2.2069(3)	2.0828(15)	2.503(4)
	2.2296(3)	2.0811(15)	2.471(7)
		2.0866(14)	
d(Si–P), Å	2.3484(3)	2.3276(14)	2.380(5)
		2.3230(13)	
C–P–C angles, °	104.76(5)–105.94(5)	106.4(3)–108.2(3)	104.2(7)–109.1(9)

Selected NMR data are shown in Table 3. In contrast to the silicon iodide complexes, the chloride and bromide complexes readily gave ^29^Si NMR spectra. In our previous study,^14^ we found that the available relaxation agents TEMPO (2,2,6,6-tetramethylpiperidineN-oxyl) and [Cr(acac)_3_] reacted with the silicon halide complexes, but in the present work, we used tris(2,2,6,6-tetramethyl-3,5-heptanedionato)chromium(III) (TMHD),^7^ which did not react with the silicon complexes.

**Table 3:** Selected Spectroscopic Data for Neutral and Cationic Silicon(IV) Phosphine Complexes

compound	^31^P{^1^H}, ppm	^29^Si, ppm^a^	^1^*J*_SiP_, Hz	ν(Si-X), cm^–1^
[SiCl_4_(PMe_3_)_2_]	2.3	–210	257	417
[SiCl_3_(PMe_3_)_2_][BAr^F^]	–3.5	–105	211	527
[SiBr_4_(PMe_3_)_2_]	–1.2	–284	227	321
[SiBr_3_(PMe_3_)_2_][BAr^F^]	3.5	–140	184	433
[SiI_3_(PMe_3_)_2_][I]	3.2			379

a Recorded in CD_2_Cl_2_ at 183 K using [Cr(TMHD)_3_] as a relaxation agent.

**Figure 5:**
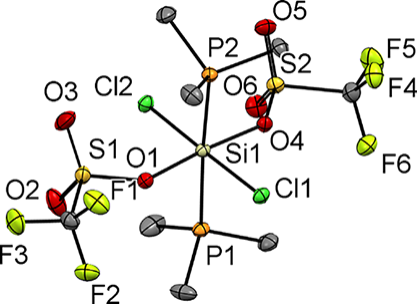
The molecular structure of [SiCl_2_(PMe_3_)_2_(OTf)_2_] showing the atom labeling scheme. Ellipsoids are drawn at the 50% probability level, and H atoms are omitted for clarity. Selected bond lengths (Å) and angles (°) are Si1–P1 = 2.3389(6), Si1–P2 = 2.3478(6), Si1–Cl1 = 2.1880(5), Si1–Cl2 = 2.1753(5), Si1–O1 = 1.8402(11), Si1–O4 = 1.8438(11), P1–Si1–P2 = 175.49(2), Cl1–Si1–Cl2 = 178.98(3), and O1–Si1–O4 = 175.01(6).

Finally, the reactions of the tetrahalides with TMSOTf (Me_3_SiO_3_SCF_3_) were also examined. The reaction of [SiCl_4_(PMe_3_)_2_] with one or two equivalents of TMSOTf in CH_2_Cl_2_ generated the six-coordinate complexes with coordinated triflate rather than lower coordinate cations, [SiCl_3_(PMe_3_)_2_(OTf)] and [SiCl_2_(PMe_3_)_2_(OTf)_2_], respectively. The same result was also found in many germanium and tin systems.^17^ Crystals of [SiCl_2_(PMe_3_)_2_(OTf)_2_] were grown by layering a CH_2_Cl_2_ solution with *n*-hexane, and the structure revealed the “all *trans*” six-coordinate complex shown in Figure 5. This is in contrast to the [GeF_2_(PMe_3_)_2_(OTf)_2_] analog, in which the triflates only interact weakly with the metal center and are mutually *cis.*^17(c)^

There is a small positive shift in the ^1^H NMR resonance upon converting the tetrachloride to the *mono*- and *bis*-triflate derivatives. However, in the ^31^P{^1^H} spectrum, there is a negative shift to δ = −0.21 for the mono-triflate and a positive shift to δ = +3.2 for the *bis*-triflate. In the case of [SiCl_2_(PMe_3_)_2_(OTf)_2_], silicon satellites are observed with^1^*J*_SiP_ = 211 Hz, smaller than for the parent tetrachloride complex.

### DFT Calculations

DFT calculations using the B3LYP-D3 functional were performed on the [SiX_3_(PMe_3_)_2_]^+^ (F, Cl, Br, I) cations in order to understand their electronic structures. For [SiCl_3_(PMe_3_)_2_]^+^ and [SiI_3_(PMe_3_)_2_]^+^, whose structures have been determined crystallographically, the geometric optimizations started from the experimental geometries, while for the [SiF_3_(PMe_3_)_2_]^+^ and [SiBr_3_(PMe_3_)_2_]^+^ cations, whose X-ray structures are not known, the geometry of [SiCl_3_(PMe_3_)_2_]^+^ was used as a starting point (with the chloride exchanged for the appropriate halide) and the structures were allowed to refine and optimize. In all cases, the calculations converged with no imaginary frequencies, showing that the optimized geometries are minima on the potential energy surface. To compare with their neutral counterparts, calculations were also performed on the tetrahalide complexes, [SiX_4_(PMe_3_)_2_] (X = F, Cl, Br, I). For X = Cl and Br, the starting geometries were based on their published crystal structures,^14^ while for X = F and I, the structures were computed starting from the geometry of [SiCl_4_(PMe_3_)_2_] (changing the Cl to the appropriate halide).

Table 4 compares the geometric parameters from the experimental X-ray crystallographic structures and the computed geometries for the [SiCl_3_(PMe_3_)_2_]^+^ and [SiI_3_(PMe_3_)_2_]^+^ cations. There is good agreement between the two (although, of course the DFT calculations assume an isolated cation, whereas the X-ray crystallographic values are determined from the solid state and include the anion and packing effects).

**Table 4:** Comparison of the Computed and Experimentally Determined Geometric Parameters for [SiCl_3_(PMe_3_)_2_]^+^ and [SiI_3_(PMe_3_)_2_]^+^

[SiCl_3_(PMe_3_)_2_]^+^	X-ray data, Å	DFT (B3LYP-D3), Å	[SiI_3_(PMe_3_)_2_]^+^	X-ray data, Å	DFT (B3LYP-D3), Å
Si–Cl	2.0828(15)	2.12782	Si–I	2.503(4)	2.57309
	2.0811(15)	2.12767		2.471(7)	2.57336
	2.0866(14)	2.12785			2.57324
Si–P	2.3272(16)	2.35909	Si–P	2.380(5)	2.44975
	2.3229(15)	2.35908			2.44975
Cl–Si–Cl	120.30(7)	120.03881	I–Si–I	119.92(14)	119.98383
	121.27(7)	119.99066		120.2(3)	120.000888
	118.42(7)	120.03881			120.01529
P–Si–P	178.97(7)	179.97953	P–Si–P	176.3(4)	179.96224

NBO analyses were performed on the computed DFT wavefunctions of the complexes to determine how the charge is distributed in the Si(IV) phosphine complexes, and the results are summarized in Table 5. In general, the positive charge on the silicon center decreases and the negative charge on X decreases upon going from F to I. The positive charge on P increases slightly. Upon going from [SiX_4_(PMe_3_)_2_] to [SiX_3_(PMe_3_)_2_]^+^, i.e., removal of an X^–^ ion, there is an increase in the charge on both the silicon center and the halide centers, except for the case of X = F, where there is a small decrease in natural charge on silicon upon fluoride abstraction. There is only a moderate decrease in the charge on the phosphorus atoms, consistent with the ^31^P{^1^H} NMR shifts being similar across the halides and also with the ^29^Si NMR shifts being much more sensitive to the specific halide present and the charge on the complex.

**Table 5:** Selected NBO Charges for Atomic Centers in Complexes [SiX_4_(PMe_3_)_2_] and [SiX_3_(PMe_3_)_2_]^+^

	[SiF_4_(PMe_3_)_2_]	[SiCl_4_(PMe_3_)_2_]	[SiBr_4_(PMe_3_)_2_]	[SiI_4_(PMe_3_)_2_]
natural charge on Si	1.81	0.77	0.49	0.14
natural charge on P	1.02	1.11	1.13	1.13
natural charge on X (av.)	–0.68	–0.48	–0.41	–0.31
	[SiF_3_(PMe_3_)_2_]^+^	[SiCl_3_(PMe_3_)_2_]^+^	[SiBr_3_(PMe_3_)_2_]^+^	[SiI_3_(PMe_3_)_2_]^+^
natural charge on Si	1.79	0.84	0.57	0.23
natural charge on P	1.00	1.07	1.10	1.08
natural charge on X (av.)	–0.64	–0.37	–0.29	–0.14

**Figure 6:**
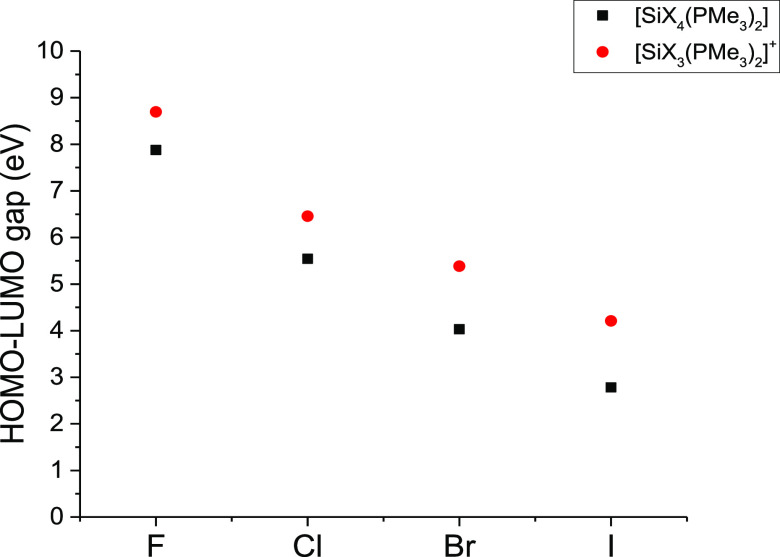
Graph showing the HOMO–LUMO energy gaps for the neutral tetrahalide and monocationic trihalide cations, determined by DFT calculations.

The HOMO–LUMO gaps of the tetrahalide and cationic complexes were investigated using DFT calculations. Going down the halide group, the HOMO–LUMO gap was found to decrease, for example, the gap for the neutral tetraiodide complexes is ca. 5 eV less than the neutral tetrafluoride. Also, for each halide type, the cation has a larger gap than the tetrahalide (see Figure 6).

## Experimental Section

Silicon halides, PMe_3_, and Et_2_P(CH_2_)_2_PEt_2_ were obtained from Strem, Alfa Aesar, or Sigma-Aldrich and used as received. TMSOTf (Sigma-Aldrich) was distilled prior to use. *o*-C_6_H_4_(PMe_2_)_2_ was made by the literature route.^18^ All reactions were conducted using Schlenk, vacuum line, and glovebox techniques and under a dry dinitrogen atmosphere. CH_2_Cl_2_ was dried by distillation from CaH_2_ and *n*-hexane, *n*-pentane and toluene from Na, and stored over activated molecular sieves. NMR solvents were also stored over 4 Å sieves.

IR spectra were recorded as Nujol mulls between CsI plates using a Perkin Elmer Spectrum 100 spectrometer over the range of 4000–200 cm^–1^. NMR spectra were recorded using a Bruker AVII 400 or AVIII HD400 spectrometer. ^1^H NMR spectra were referenced to residual solvent resonances,^19^F{^1^H} NMR spectra to external CFCl_3_, ^31^P{^1^H} NMR spectra to aqueous 85% H_3_PO_4_, and ^29^Si NMR spectra to TMS. The latter used tris(2,2,6,6-tetramethyl-3,5-heptanedionato)chromium(III) (TMD) as a relaxation agent. Microanalytical measurements were performed by Medac Ltd.

### X-Ray Crystallography

Single crystals were grown as described. Single-crystal X-ray data were collected using a Rigaku AFC12 goniometer equipped with an enhanced-sensitivity (HG) Saturn724+ detector mounted at the window of an FR-E+ SuperBright molybdenum (λ = 0.71073 Å) rotating anode generator with VHF or HF Varimax optics (70 or 100 μm focus), with the crystal held at 100 K (N_2_ Cryostream). Structure refinements were performed with either SHELX(S/L)97 or SHELX(S/L)2013, through Olex225^19^ and were mostly straightforward, with H atoms bonding to C atoms placed in calculated positions using default C–H distances. Where additional constraints or restraints were required, details are provided in the cif file for each structure. For [SiI_4_{Et_2_P(CH_2_)_2_PEt_2_}], the crystal was twinned, and the structure was refined against a deconvoluted data set. For [SiI_3_(PMe_3_)_2_][I], there were alerts due to residual election density near the iodine atoms, most likely due to absorption, as well as some residual disordered solvent in the central channel, which could not be accounted for by applying a mask. CCDC reference numbers for the crystallographic information files in cif format are [SiI_4_{Et_2_P(CH_2_)_2_PEt_2_}] 2162654, [SiI_4_{*o*-C_6_H_4_(PMe_2_)_2_}] 2162655, [SiCl_3_(PMe_3_)_2_][BAr^F^] 2162656, [SiI_3_(PMe_3_)_2_][I] 2162657, and [SiCl_2_(PMe_3_)_2_(OTf)_2_] 2162658.

The structure of [SiI_3_(PMe_3_)_2_][I]·CH_2_Cl_2_·0.5C_6_H_14_ shows channels through the extended lattice. The residual electron density within the enclosed voids is attributed to disordered *n*-hexane, which could not be satisfactorily modeled, so a solvent mask was used. For this structure, there are 150e- unaccounted for in two enclosed voids of 210.7 Å^3^, which is consistent with three *n*-hexane molecules per unit cell (the second type of channel voids corresponds to a volume 426 Å^3^ per unit cell and is empty). Similarly, for the structure of [SiI_4_{*o*-C_6_H_4_(PMe_2_)_2_}]·0.5C_6_H_14_, there are channels through the extended lattice, which also appear to contain disordered *n*-hexane. For this structure, there are 100e- unaccounted for in a void of 250 Å^3^, which is consistent with two *n*-hexane molecules per unit cell. A solvent mask was also used here. For [SiI_4_(PEt_2_{CH_2_}_2_PEt_2_)], the crystal was twinned and the resulting structure was refined against deconvoluted data from a single component.

### Complex Syntheses

#### [SiI_3_(PMe_3_)_2_][I]

SiI_4_ (0.500 g, 0.99 mmol) was dissolved *n*-hexane (5 mL), and to this, PMe_3_ (0.142 g, 1.85 mmol) was added as a solution in *n*-hexane (3 mL), causing a yellow powder to precipitate immediately. The reaction mixture was stirred for 1 h. The supernatant was filtered away, and the solid was washed with *n*-hexane (3 × 10 mL) and dried in vacuo to yield a yellow solid, with yield of 0.478 g (75%). Crystals of this complex suitable for X-ray crystallography were grown by layering a CH_2_Cl_2_ solution of the complex with *n*-hexane. The complex can also be isolated using *n*-pentane as solvent. Multiple attempts to obtain a good microanalysis on samples of this complex were unsuccessful due to the presence of varying amounts of *n*-hexane, which was also evident in the ^1^H NMR spectrum and in the X-ray crystal structure. Anal. Calc. for C_6_H_18_I_4_P_2_Si·1/4C_6_H_14_ (709.40): C, 12.7; H, 3.1. Found: C, 11.8; H, 3.6%. IR (Nujol/cm^–1^): ν = 379 s (Si–I). ^1^H NMR (CD_2_Cl_2_, 298 K): δ = 1.66 (d,^1^*J*_PH_ = 11 Hz, CH_3_). ^31^P{^1^H} NMR (CD_2_Cl_2_, 298 K): δ = 3.2 (br s); (253 K): δ = 4.6 (s,^1^*J*_SiP_ = 93 Hz). ^29^Si NMR (CD_2_Cl_2_, 253 K): a weak and poorly resolved triplet tentatively assigned to the complex was observed at −233 ppm.

#### [SiI_4_{*o*-C_6_H_4_(PMe_2_)_2_}]

SiI_4_ (0.150 g, 0.28 mmol) was dissolved in *n*-hexane. To this, *o*-C_6_H_4_(PMe_2_)_2_ (0.055 g, 0.28 mmol) was added as a solution in *n*-hexane (3 mL), causing immediate precipitation of an orange powder. The reaction mixture was stirred for 1 h. The supernatant was filtered away, and the solid was washed with *n*-hexane (3 × 10 mL) and dried in vacuo to yield an orange solid, with yield of 0.173 g (85%). Crystals of this complex suitable for single crystal X-ray analysis were grown by layering a CH_2_Cl_2_ solution of the complex with *n*-hexane. The complex can also be synthesized using *n*-pentane as solvent. Anal. Calc. for C_10_H_16_I_4_P_2_Si·1/2C_5_H_12_ (769.94): C, 19.5; H, 2.9 Found: C, 19.3; H, 2.7%. IR (Nujol/cm^–1^): *ν* = 281 m, 310 m (Si–I) ^1^H NMR (CD_2_Cl_2_, 298 K): δ = 1.99 (m, [12H], CH_3_), 7.69–7.90 (m, [4H], ArH). ^31^P{^1^H} NMR (CD_2_Cl_2_, 298 K): δ = −33.6 (br s,^1^*J*_SiP_ = not resolved); (253 K): δ = −32.1 (br,^1^*J*_SiP_ not resolved). ^29^Si NMR (CD_2_Cl_2_, 298/253 K): not observed.

#### [SiI_4_{Et_2_P(CH_2_)_2_PEt_2_}]

SiI_4_ (0.300 g, 5.60 mmol) was dissolved in toluene (5 mL); to this, Et_2_P(CH_2_)_2_PEt_2_ (0.116 g, 5.62 mmol) was added as a solution in toluene (2 mL), causing the precipitation of an orange solid. The reaction was stirred for 1 h. The supernatant was filtered away, and the solid was washed with *n*-hexane (3 × 10 mL) and dried in vacuo to yield an orange solid. Crystals of the complex were grown by layering a CH_2_Cl_2_ solution of the complex with *n*-hexane. The complex can also be synthesized using *n*-pentane as solvent. Yield: 0.233 g (56%). Anal. Calc. for C_10_H_24_I_4_P_2_Si·1/2C_5_H_12_ (778): C, 19.3; H, 3.9. Found: C, 19.2; H, 4.3%. IR (Nujol/cm^–1^): ν = 278 m, 305 m (Si–I). ^1^H NMR (CD_2_Cl_2_, 298 K): δ = 1.41 (m, [12H], CH_3_), 1.98 (m, [4H], CH_2_), 2.36 (br m, [8H], CH_2_). ^31^P{^1^H} NMR (CD_2_Cl_2_, 298 K): δ = −25.6 (br s); (183 K): δ = −20.8 (br, s). ^29^Si NMR (CD_2_Cl_2_, 183 K): a weak and poorly resolved triplet tentatively assigned to this complex was observed at −696 ppm.

#### [SiCl_3_(PMe_3_)_2_][BAr^F^]

[SiCl_4_(PMe_3_)_2_] (0.200 g, 0.62 mmol) was dissolved in CH_2_Cl_2_ (5 mL), and Na[BAr^F^] (0.550 g, 0.62 mmol) was added as a solid. The reaction mixture was stirred for 1 h during which a small amount of white precipitate formed (NaCl). The supernatant was filtered away and concentrated in vacuo to yield a white solid. Crystals of the complex were grown by layering a CH_2_Cl_2_ solution of the complex with *n*-hexane. Yield: 0.492 g (69%). Anal. Calc. for C_38_H_30_BCl_3_F_24_P_2_Si (1149.73): C, 39.7; H, 2.6. Found: C, 39.7; H, 3.1%. IR (Nujol/cm^–1^): ν = 527 s (Si–Cl).^1^H NMR (CD_2_Cl_2_, 298 K): δ = 1.60 (m, [18H], CH_3_), 7.57 (s, [4H], ArH), 7.73 (s, [8H], ArH). ^31^P{^1^H} NMR (CD_2_Cl_2_, 298 K): δ = −3.5 (^1^*J*_SiP_ = 211 Hz); (183 K): δ = −2.4 (^1^*J*_SiP_ = 211 Hz). ^29^Si NMR (CD_2_Cl_2_, 298 K): δ = −104 (t,^1^*J*_SiP_ = 211 Hz); (183 K): −105 (t,^1^*J*_SiP_ = 211 Hz).

#### [SiBr_3_(PMe_3_)_2_][BAr^F^]

[SiBr_4_(PMe_3_)_2_] (0.100 g, 0.20 mmol) was dissolved in CH_2_Cl_2_ (5 mL) to which Na[BAr^F^] (0.177 g, 0.20 mmol) was added as a solid, and the reaction mixture was stirred for 1 h, during which a small amount of white precipitate formed, which was removed by filtration (NaBr). The supernatant was concentrated in vacuo to yield a white solid. Yield: 0.233 g (91%). Anal. Calc. for C_38_H_30_BBr_3_F_24_P_2_Si (1283.11): C, 35.6; H, 2.4. Found: C, 35.8; H, 2.7%. IR (Nujol/cm^–1^): 433 s (Si–Br). ^1^H NMR (CD_2_Cl_2_, 298 K): δ = 1.62 (m, [18H], CH_3_), 7.57 (s, [4H], ArH), 7.73 (s, [8H], Ar–H). ^31^P{^1^H} NMR (CD_2_Cl_2_, 298 K): δ = +3.5 (br s); (183 K): δ = +6.3 (s,^1^*J*_SiP_ = 184 Hz). ^29^Si NMR (CD_2_Cl_2_, 298 K): not observed; (183 K): −140 (t,^1^*J*_SiP_ = 184 Hz).

#### [SiCl_3_(PMe_3_)_2_(OTf)]

[SiCl_4_(PMe_3_)_2_] (0.100 g, 0.31 mmol) was dissolved in CH_2_Cl_2_ (5 mL), and TMSOTf (0.069, 0.31 mmol) was added as a solution in CH_2_Cl_2_ (2 mL), resulting in a colorless solution. The reaction was stirred for 1 h, after which the volatiles were removed in vacuo to yield a white solid, which was washed with *n*-hexane (3 × 10 mL) and dried in vacuo. Yield 0.119 g (88%). Anal. Calc. for C_7_H_18_Cl_3_F_3_O_3_P_2_SSi (435.63): C, 19.3; H, 4.2. Found: C 19.3; H, 4.4%. IR (Nujol/cm^–1^): ν = 383 m, 423 m (Si–Cl). ^1^H NMR (CD_2_Cl_2_, 298 K): δ = 1.67 (d,^2^*J*_PH_ = 12 Hz, CH_3_). ^31^P{^1^H} NMR (CD_2_Cl_2_, 298 K): δ = −0.21 (s,^1^*J*_SiP_ = 248 Hz). ^29^Si NMR (CD_2_Cl_2_, 298 K): −146 (t,^1^*J*_SiP_ = 248 Hz).

#### [SiCl_2_(PMe_3_)_2_(OTf)_2_]

[SiCl_4_(PMe_3_)_2_] (0.200 g, 0.62 mmol) was dissolved in CH_2_Cl_2_ (5 mL) to which TMSOTf (0.216 g, 1.24 mmol) was added as a solution in CH_2_Cl_2_ (2 mL), resulting in a colorless solution. The reaction was stirred for 1 h, and the reaction mixture was layered with *n*-hexane (10 mL) and stored at −18 °C, which after 1 day afforded a crop of colorless crystals, which were collected by filtration, washed with hexane (3 × 10 mL), and dried in vacuo. Yield: 0.231 g (68%). Anal. Calc. for C_8_H_18_Cl_2_F_6_O_6_P_2_S_2_Si·1/4C_6_H_14_ (570.79): C, 20.0; H, 3.8. Found: C, 19.6; H, 4.2%. IR (Nujol/cm^–1^): 442 m, 462 m (Si–Cl). ^1^H NMR (CD_2_Cl_2_, 298 K): δ = 1.67 (d,^2^*J*_PH_ = 12 Hz, CH_3_). ^31^P{^1^H} NMR (CD_2_Cl_2_, 298 K): δ = 3.2 (s,^1^*J*_SiP_ = 311 Hz). ^29^Si NMR (CD_2_Cl_2_, 298 K): −194 (t,^1^*J*_SiP_ = 311 Hz).

### DFT Computational Details

The electronic structures of the isolated molecules/cations were investigated using DFT calculations using the Gaussian 16 W software package.^20^ The density functional chosen was B3LYP-D3,^21^ corrected for dispersion,^20^ using the basis set 6-311G(d)^22^ for all atoms, except for the heavy Br and I atoms, where the pseudo-potential Lanl2dz basis sets were used (with the previously mentioned basis set used for the lighter atoms).^23^ For [SiCl_4_(PMe_3_)_2_], [SiBr_4_(PMe_3_)_2_], [SiI_3_(PMe_3_)_2_]^+^, and [SiCl_3_(PMe_3_)_2_]^+^, the initial geometries were taken from their crystal structures; prior to geometry optimization For [SiF_4_(PMe_3_)_2_] and [SiI_4_(PMe_3_)_2_], the structure of the complex [SiCl_4_(PMe_3_)_2_] was chosen as the starting geometry, with the halide atoms exchanged as appropriate. For [SiF_3_(PMe_3_)_2_]^+^ and [SiBr_3_(PMe_3_)_2_]^+^, the [SiCl_3_(PMe_3_)_2_]^+^ cation was chosen as the starting geometry with the halide atoms exchanged as appropriate. In all cases, the geometry optimization converged to a stable geometry with no imaginary frequencies. For the complexes with known X-ray crystal structures, the DFT-computed geometries were compared with the crystallographic geometries and were found to be in good agreement.

## Conclusions

The first examples of phosphine complexes of the weakly Lewis acidic silicon tetraiodide have been characterized, including the spontaneous formation of the cation [SiI_3_(PMe_3_)_2_][I], which contrasts with the chemistry of the lighter halides with PMe_3_, where only the neutral *trans*-octahedral [SnX_4_(PMe_3_)_2_] complexes were observed.^14^ Silicon(IV) iodide was also shown to react with the diphosphine ligands Et_2_P(CH_2_)_2_PEt_2_ and *o*-C_6_H_4_(PMe_2_)_2_ to form six-coordinate neutral complexes with *cis*-octahedral coordination, the first known soft donor complexes featuring the SiI_4_ unit.

There are no structurally characterized silicon tetraiodide complexes with neutral As-donor or S-donor ligands,^2,11^ and with NHC ligands, all structurally characterized examples are cationic due to iodide displacement.^5,9,10^

The discrete [SiX_3_(PMe_3_)_2_]^+^ (X = Cl, Br) cations have been synthesized by the reaction of the tetrahalide complexes with Na[BAr^F^], while the reaction of [SiCl_4_(PMe_3_)_2_] with TMSOTf leads to the formation of neutral complexes [SiCl_3_(PMe_3_)_2_(OTf)] and [SiCl_2_(PMe_3_)_2_(OTf)_2_], in which the OTf groups remain coordinated to the silicon center. The reactions of [SiX_4_(PMe_3_)_2_] with AlX_3_ led to the removal of the phosphine from the silicon center. Such Si(IV) cations may find application for Lewis acid promoted transformations in organic chemistry.

DFT calculations suggest that the charge on the silicon center in [SiX_4_(PMe_3_)_2_] and [SiX_3_(PMe_3_)_2_]^+^ increases in the series F > Cl > Br > I, in line with expectations based on the decreasing electronegativity going down group 17. Upon going from the tetrahalide species to the trihalide cations, NBO analysis shows that the majority of the change in the charge occurs in the atoms of the ‘SiX_3_’ fragment with no significant change in the charge on the phosphorus atom.

## Supporting Information Available

Crystallographic parameters for the crystal structures reported; cif files for the crystal structures described; full details of the computational work; and original IR, ^1^H, ^19^F{^1^H}, ^29^Si, and ^31^P{^1^H} NMR spectra for the reported complexes (PDF)
