# Supplementary material for: Neutral and Cationic Complexes of Silicon(IV) Halides with Phosphine Ligands
Source: Inorg Chem. 2022 Oct 12;61(42):16905–13. doi: 10.1021/acs.inorgchem.2c02949 (PMC9597660; doi:10.1021/acs.inorgchem.2c02949)
Supplement: Supplementary file 1 — ic2c02949_si_002.pdf [file ic2c02949_si_002.pdf]

Supporting information for

Neutral and cationic complexes of silicon(IV) halides with phosphine ligands.

Rhys P. King[a], John M. Dyke[a], William Levason[a] and Gillian Reid[a]

[a]School of Chemistry, University of Southampton, Southampton SO17 1BJ, UK; email: G.Reid@soton.ac.uk

Table S1 X-ray crystallographic data.

Compound	$[SiI_3(PMe_3)_2][I]\cdot CH_2Cl_2\cdot 0.5\ C_6H_{14}$	$[SiI_4\{o\text{-}C_6H_4(PMe_2)_2\}]\cdot 0.5C_6H_{14}$	$[SiI_4\{Et_2P(CH_2)_2PEt_2\}]$	$[SiCl_3(PMe_3)_2][BAr^F]$	$[SiCl_2(PMe_3)_2(OTf)_2]$
Formula	$C_{10}H_{27}Cl_2I_4P_2Si$	$C_{13}H_{23}I_4P_2Si$	$C_{10}H_{24}I_4P_2Si$	$C_{38}H_{30}BCl_3F_{24}P_2Si$	$C_8H_{18}C_{12}F_6O_6P_2S_2Si$
M	815.84	776.978	741.953	1149.81	549.289
Crystal system	hexagonal	monoclinic	monoclinic	orthorhombic	monoclinic
Space group (no.)	$P6_3/mmc$ (194)	$P2_1/n$ (14)	Cc (9)	Pbca (61)	$P2_1$ (4)
a /Å	18.1858(3)	7.22644(14)	12.8535(6)	17.7948(7)	7.98577(19)
b /Å	18.1858(3)	16.7515(3)	8.8973(3)	19.5965(6)	12.3268(3)
c /Å	13.4510(3)	17.0301(4)	17.8528(9)	26.8774(11)	10.7399(2)
α /°	90	90	90	90	90
β /°	90	101.887(2)	103.382(5)	90	92.284(2)
γ /°	120	90	90	90	90
U /Å^3	3852.56(15)	2017.35(7)	1986.24(16)	9372.6(6)	1056.39(4)
Z	6	4	4	8	2
μ(Mo-K$_α$) /mm^{-1}	5.223	6.387	6.480	0.415	0.787
F(000)	2262	1423	1355	4592	558
Total no. reflns	167082	52053	10249	66260	29360
R_{int}	0.094	0.079	0.093	0.063	0.041
Unique reflns	2407	6706	3943	14048	6701
No. of params, restraints	52, 0	159, 0	158, 62	626, 3	250, 1
GOF	1.079	1.084	1.013	1.012	1.044
R_1, wR_2 [$I > 2\sigma(I)$][b]	0.097, 0.240	0.026, 0.059	0.079, 0.213	0.070, 0.180	0.026, 0.059
R_1, wR_2 (all data)	0.116, 0.252	0.029, 0.060	0.082, 0.215	0.138, 0.237	0.030, 0.060

[a] Common items: T = 100 K; wavelength (Mo-K$_α$) = 0.71073 Å; θ(max) = 27.5°; [b] $R_1 = \Sigma||F_o|-|F_c||/\Sigma|F_o|$; $wR_2=[\Sigma w(F_o^2-F_c^2)_2/\Sigma wF_o^4]^{1/2}$

Spectroscopic Data

Figure S1 [$SiCl_4(PMe_3)_2$]

Figure S1(a) ^1H NMR spectrum of [$SiCl_4(PMe_3)_2$] (CD_2Cl_2, 298 K)

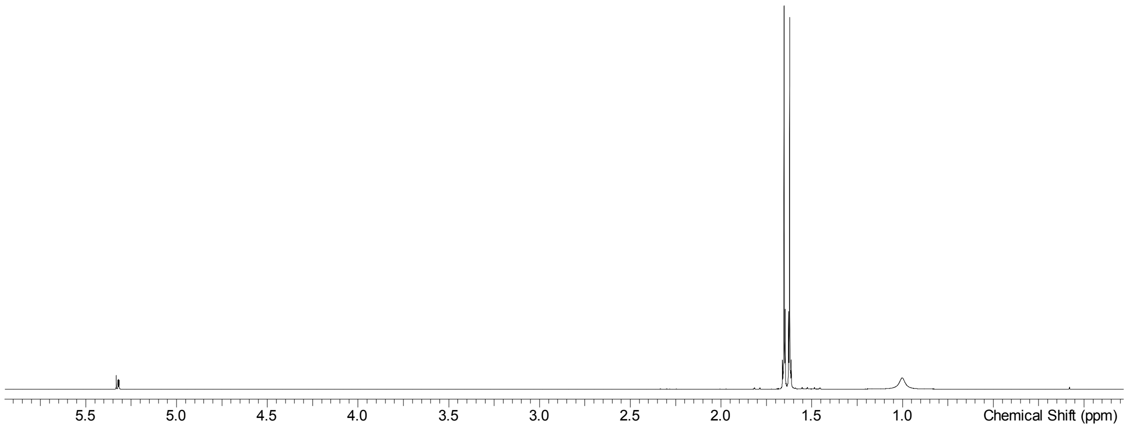

Figure S1(b) $^{31}P\{^1H\}$ NMR spectrum of [$SiCl_4(PMe_3)_2$] (CD_2Cl_2, 298 K)

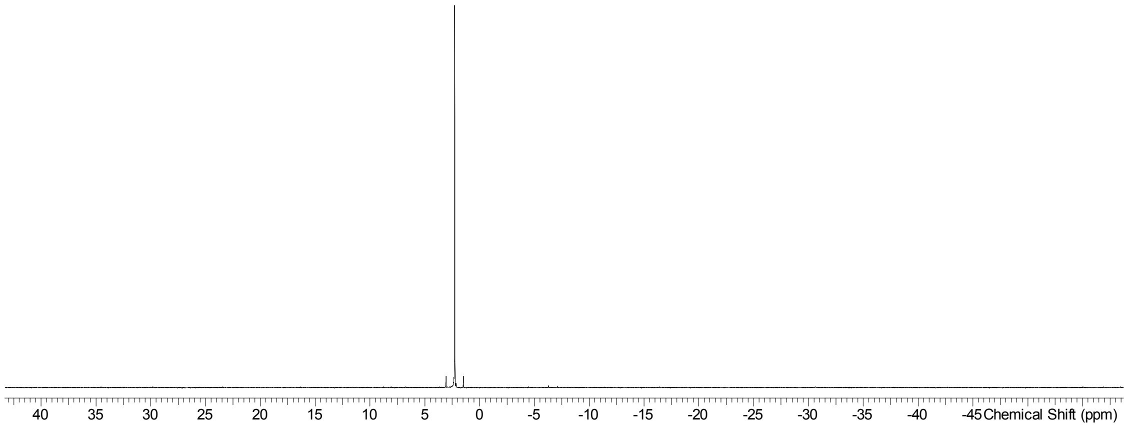

Figure S1(c) ^{29}Si NMR spectrum of [$SiCl_4(PMe_3)_2$] (CD_2Cl_2, 298 K)

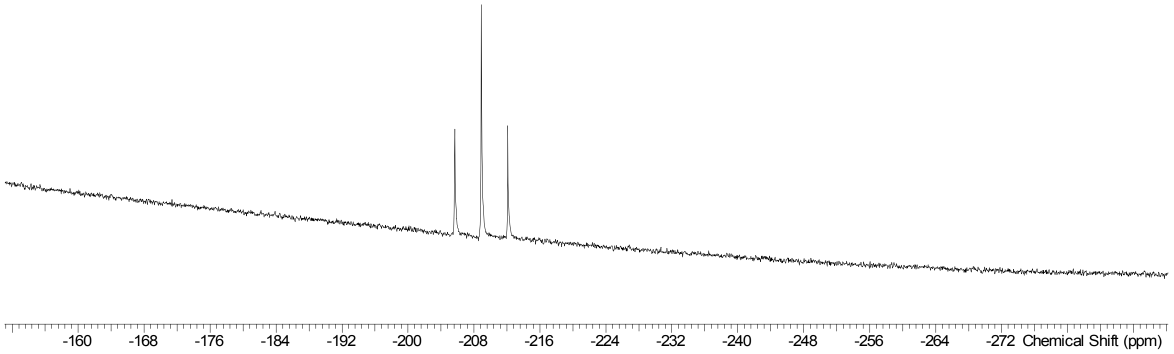

Figure S1(d) ^{29}Si NMR spectrum of $[SiCl_4(PMe_3)_2]$ (CD_2Cl_2, 183 K)

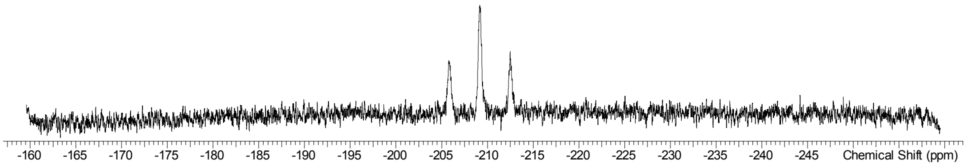

Figure S2 $[SiBr_4(PMe_3)_2]$

Figure S2(a) ^{1}H NMR spectrum of $[SiBr_4(PMe_3)_2]$ (CD_2Cl_2, 298 K)

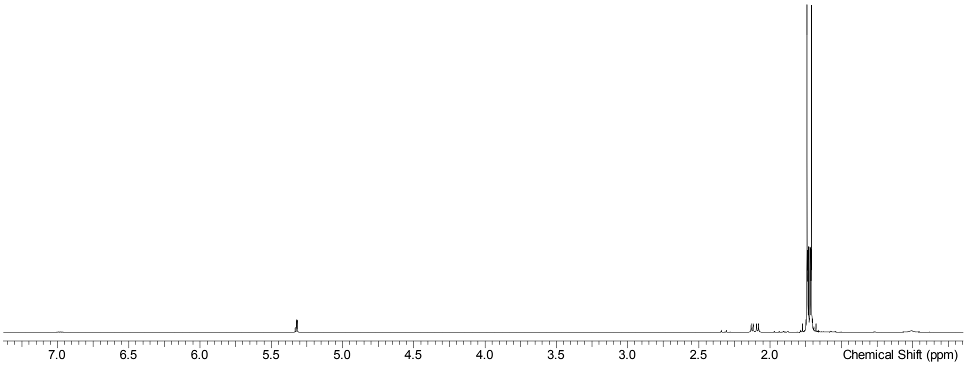

Figure S2(b) $^{31}P\{^{1}H\}$ NMR spectrum of $[SiBr_4(PMe_3)_2]$ (CD_2Cl_2. 298 K)

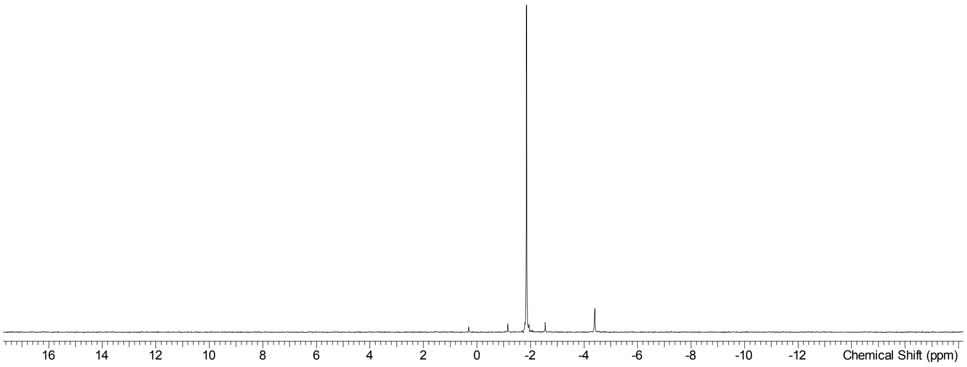

Figure S2(c) ^{29}Si NMR spectrum of $[SiBr_4(PMe_3)_2]$ (CD_2Cl_2. 298 K)

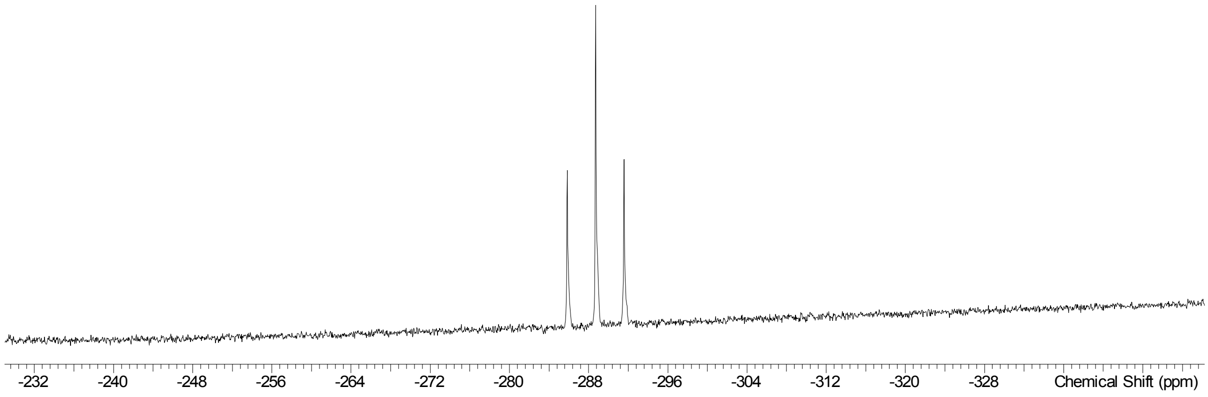

Figure S2(d) ^{29}Si NMR spectrum of $[SiBr_4(PMe_3)_2]$ (CD_2Cl_2. 193 K)

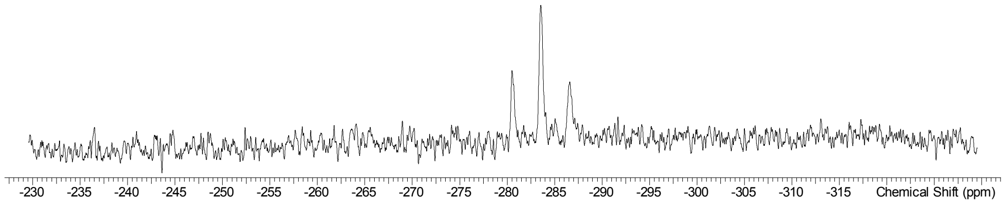

Figure S3 $[SiI_3(PMe_3)_2][I]$

Figure S3(a) ^{1}H NMR spectrum of $[SiI_3(PMe_3)_2][I]$ (CD_2Cl_2, 298 K) * $[HPMe_3]^+$

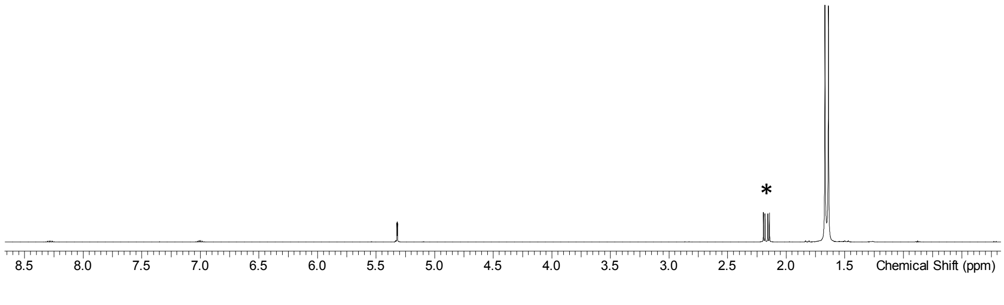

Figure S3(b) $^{31}P\{^{1}H\}$ NMR spectrum of $[SiI_3(PMe_3)_2][I]$ (CD_2Cl_2, 298 K) * $[HPMe_3]^+$

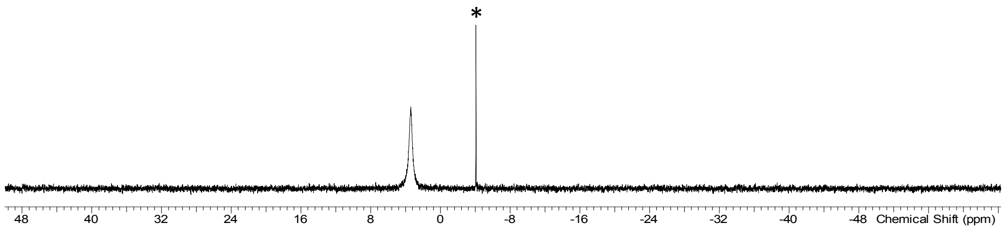

Figure S3(c) $^{31}P\{^{1}H\}$ NMR spectrum of $[SiI_3(PMe_3)_2][I]$ (CD_2Cl_2, 253 K) * $[HPMe_3]^+$

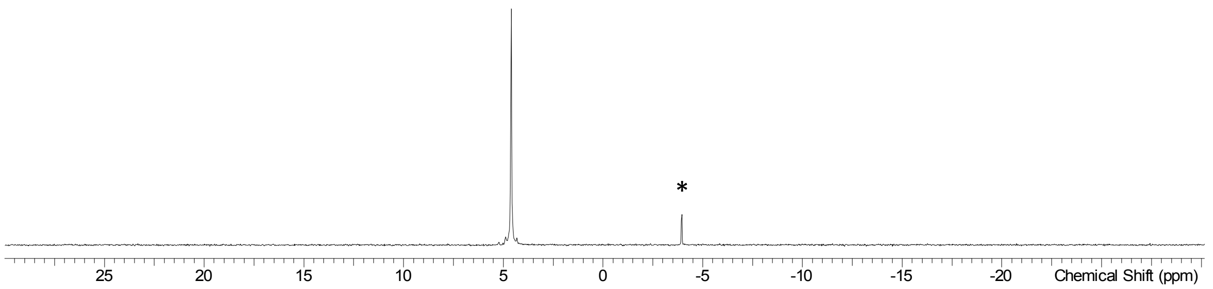

Figure S3(d) IR spectrum of $[SiI_3(PMe_3)_2][I]$ (Nujol/cm^{-1})

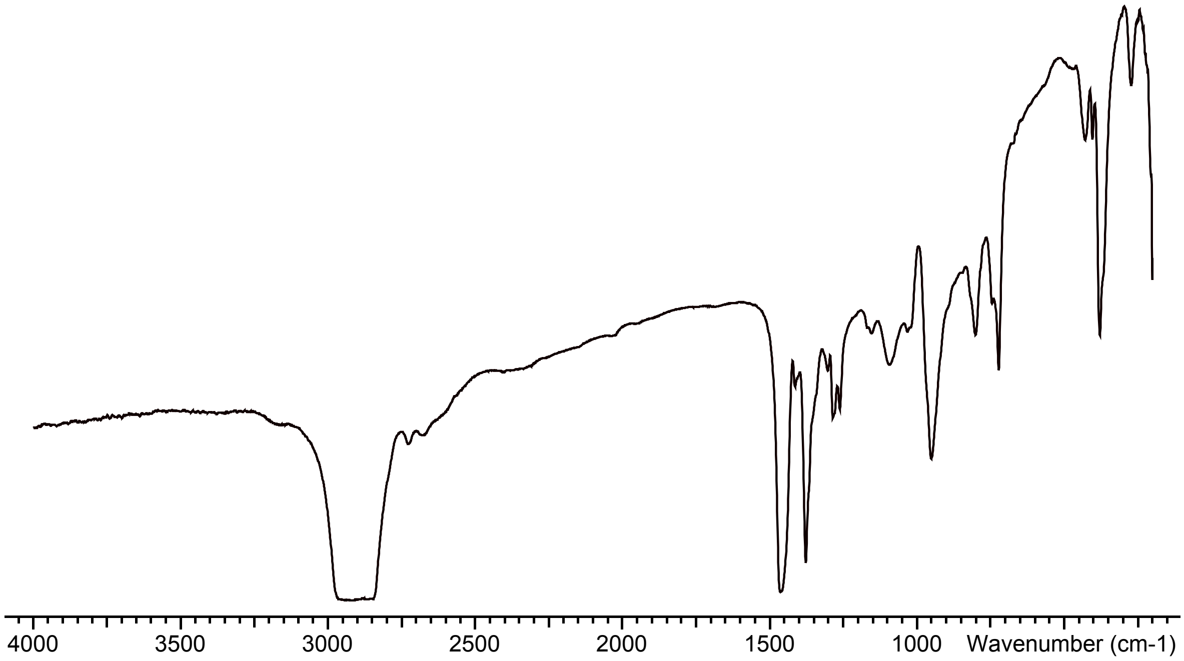

Figure S4 [$SiI_4(o\text{-}C_6H_4(PMe_2)_2)$]

Figure S4(a) ^1H NMR spectrum of [$SiI_4(o\text{-}C_6H_4(PMe_2)_2)$] ($CD_2Cl_2$, 298 K)

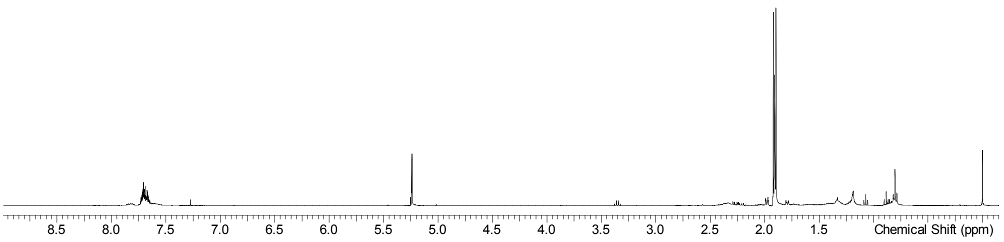

Figure S4(b) $^{31}P\{^1H\}$ NMR spectrum of [$SiI_4(o\text{-}C_6H_4(PMe_2)_2)$] ($CD_2Cl_2$, 298 K)

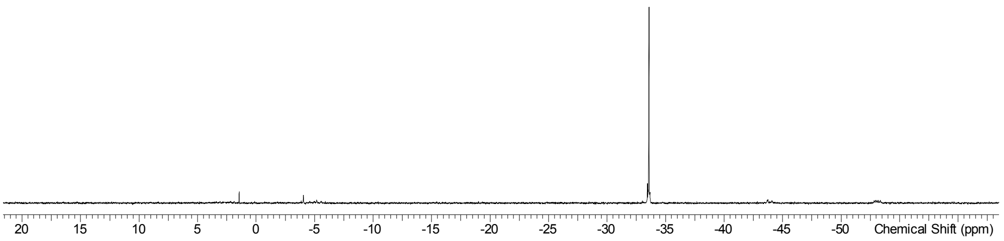

Figure S4(c) $^{31}P\{^1H\}$ NMR spectrum of [$SiI_4(o\text{-}C_6H_4(PMe_2)_2)$] ($CD_2Cl_2$, 253 K)

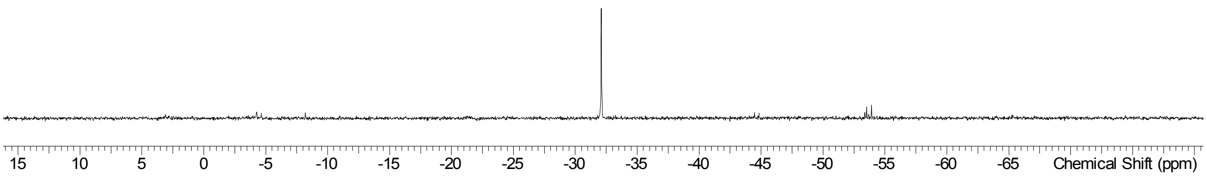

Figure S4(d) IR spectrum of [SiI_4(*o*-$C_6H_4(PMe_2)_2$)] (Nujol/cm^{-1})

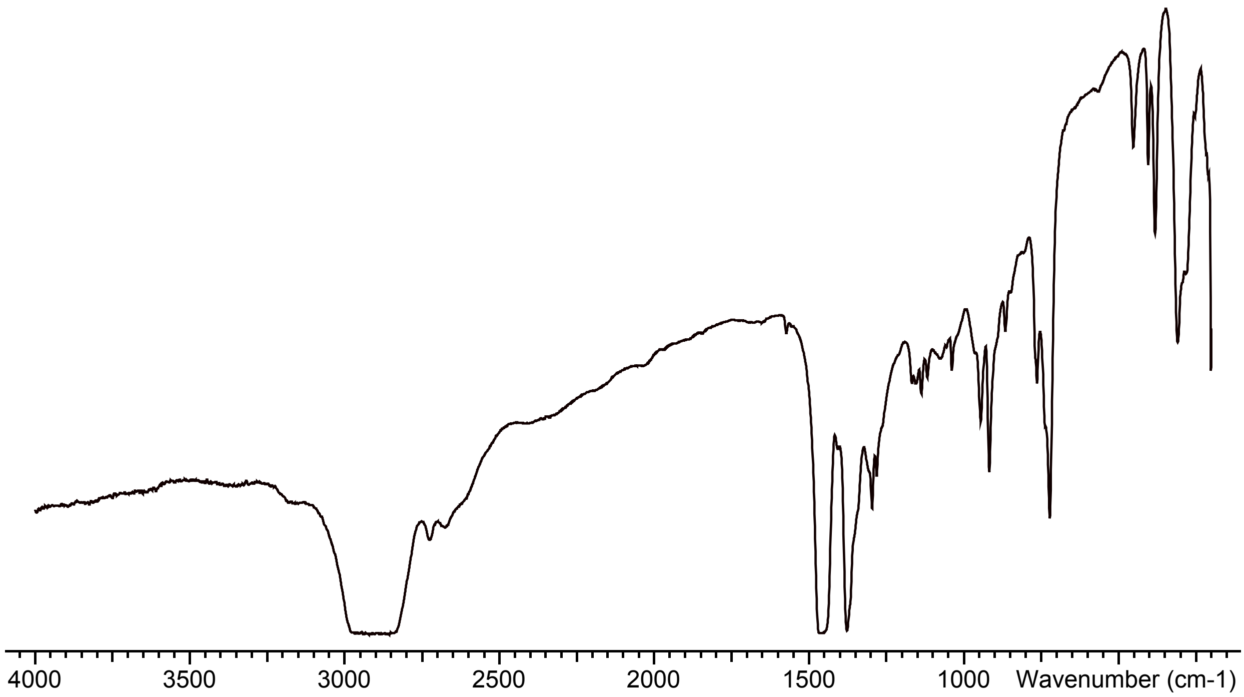

Figure S5 [SiI_4(depe)]

Figure S5(a) ^{1}H NMR spectrum of [SiI_4(depe)] (CD_2Cl_2, 298 K), * = monoprotonated ligand

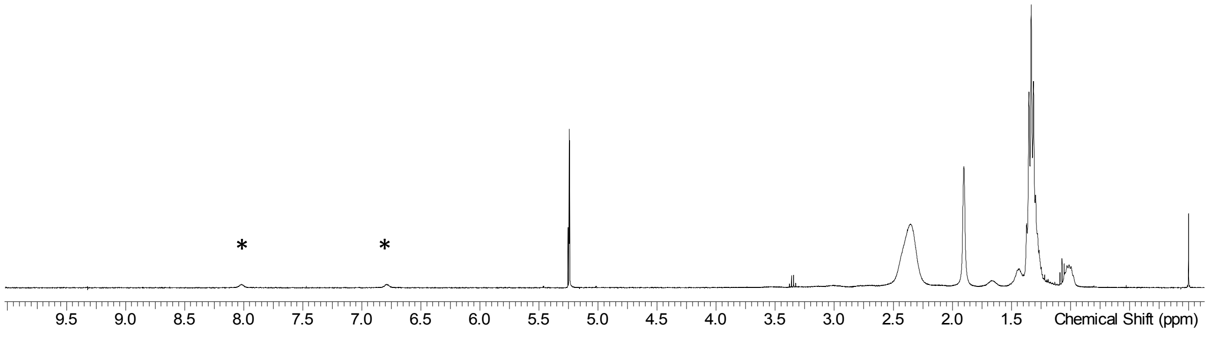

Figure S5(b) $^{31}P\{^1H\}$ NMR spectrum of $[SiI_4(depe)]$ (CD_2Cl_2, 298 K), * = monoprotonated ligand

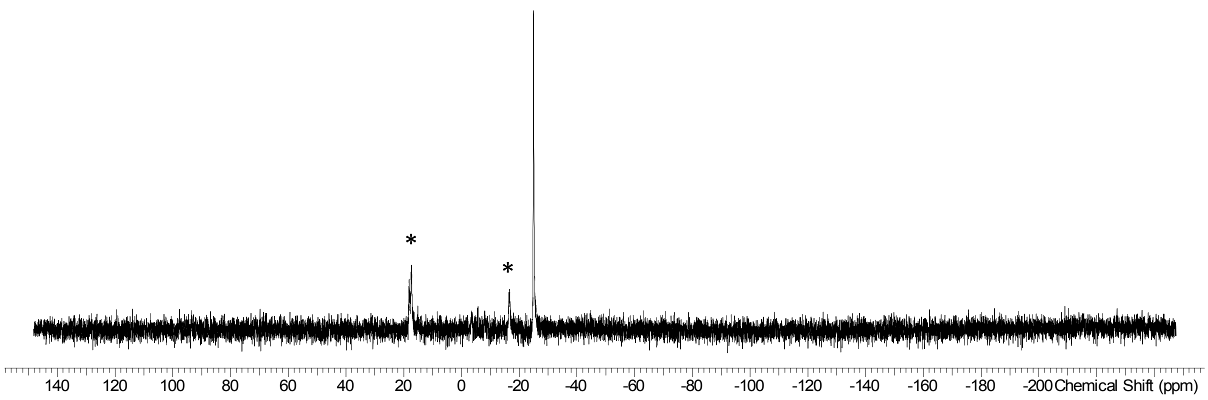

Figure S5(c) $^{31}P\{^1H\}$ NMR spectrum of $[SiI_4(depe)]$ (CD_2Cl_2, 183 K)

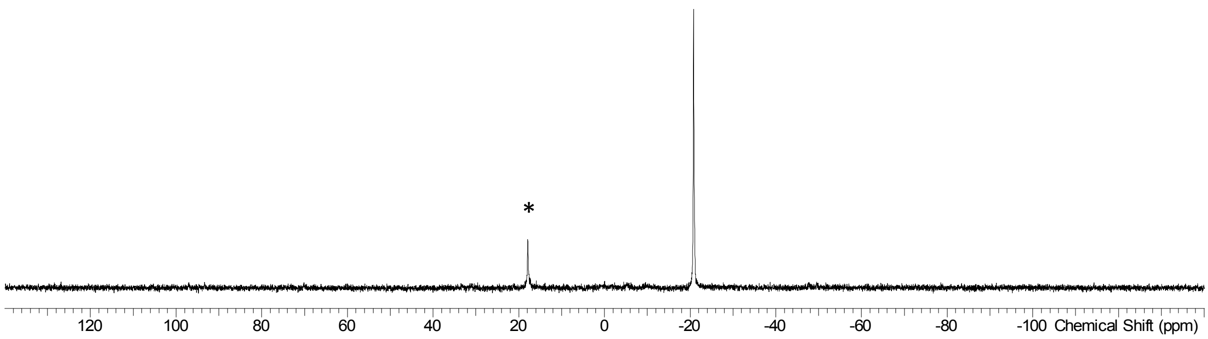

Figure S5(d) IR spectrum of $[SiI_4(depe)]$ (Nujol/cm^{-1})

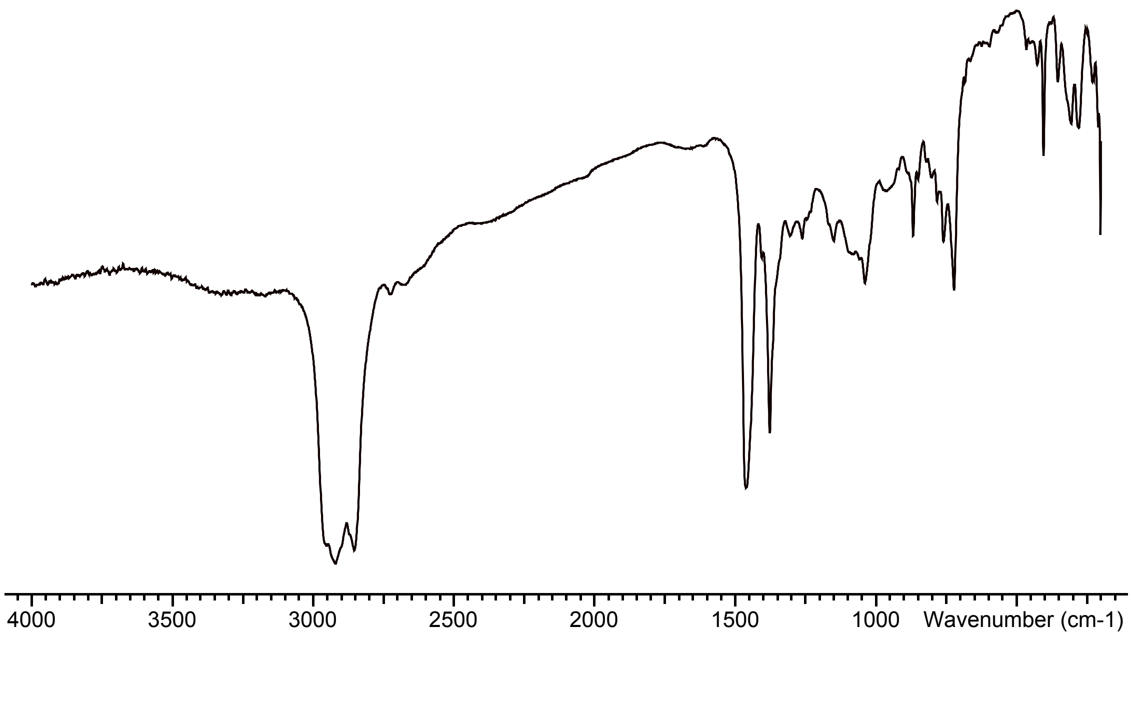

Figure S6 [$SiCl_3(PMe_3)_2$][BAr^F]

Figure S6(a) ^1H NMR spectrum of [$SiCl_3(PMe_3)_2$][BAr^F] (CD_2Cl_2, 298 K)

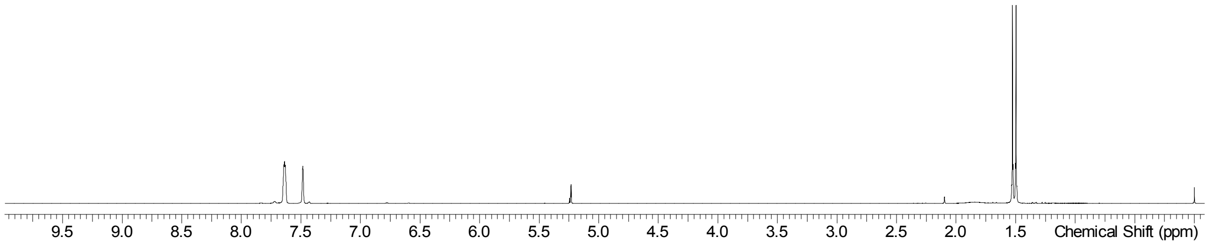

Figure S6(b) $^{31}P\{^1H\}$ NMR spectrum of [$SiCl_3(PMe_3)_2$][BAr^F] (CD_2Cl_2, 298 K)

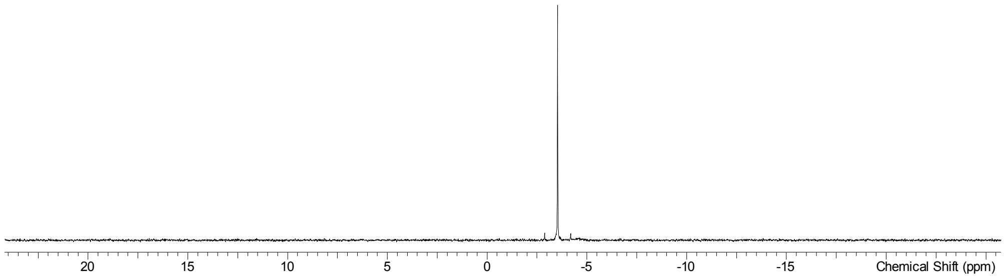

Figure S6(c) $^{31}P\{^1H\}$ NMR spectrum of [$SiCl_3(PMe_3)_2$][BAr^F] (CD_2Cl_2, 183 K)

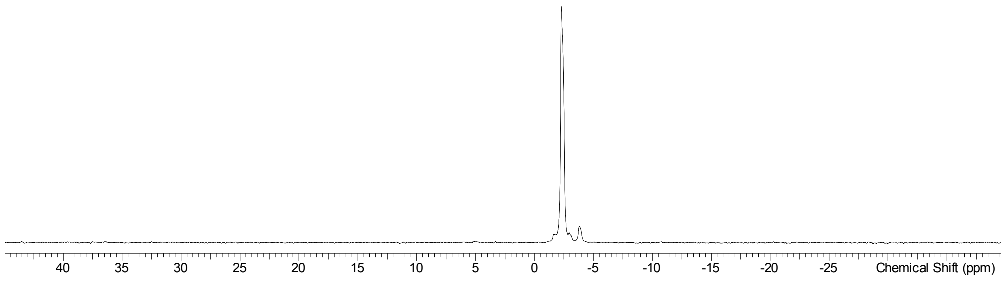

Figure S6(d) ^{29}Si NMR spectrum of $[SiCl_3(PMe_3)_2][BAr^F]$ (CD_2Cl_2, 298 K), * = $SiCl_4$ impurity

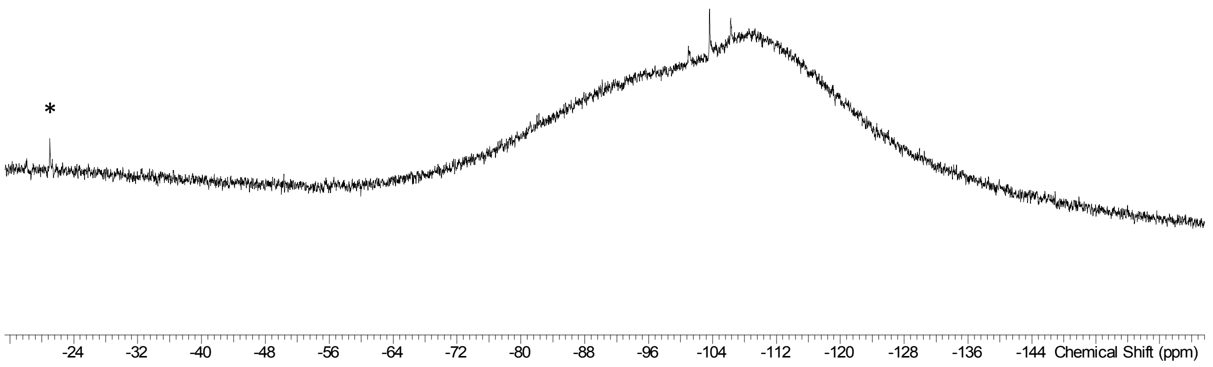

Figure S6(e) ^{29}Si NMR spectrum of $[SiCl_3(PMe_3)_2][BAr^F]$ (CD_2Cl_2, 183 K)

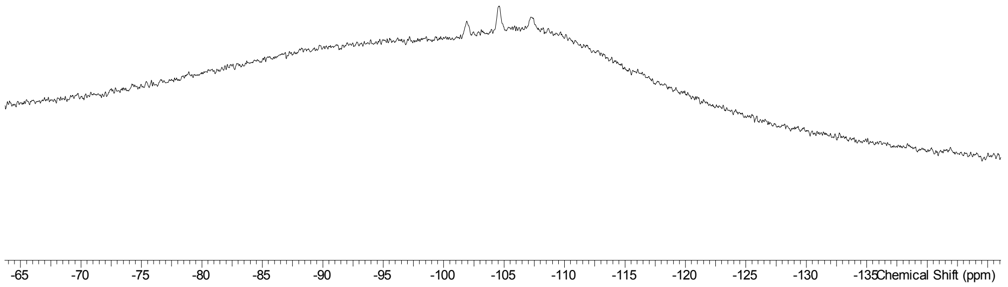

Figure S6(f) IR spectrum of $[SiCl_3(PMe_3)_2][BAr^F]$ (Nujol/cm^{-1})

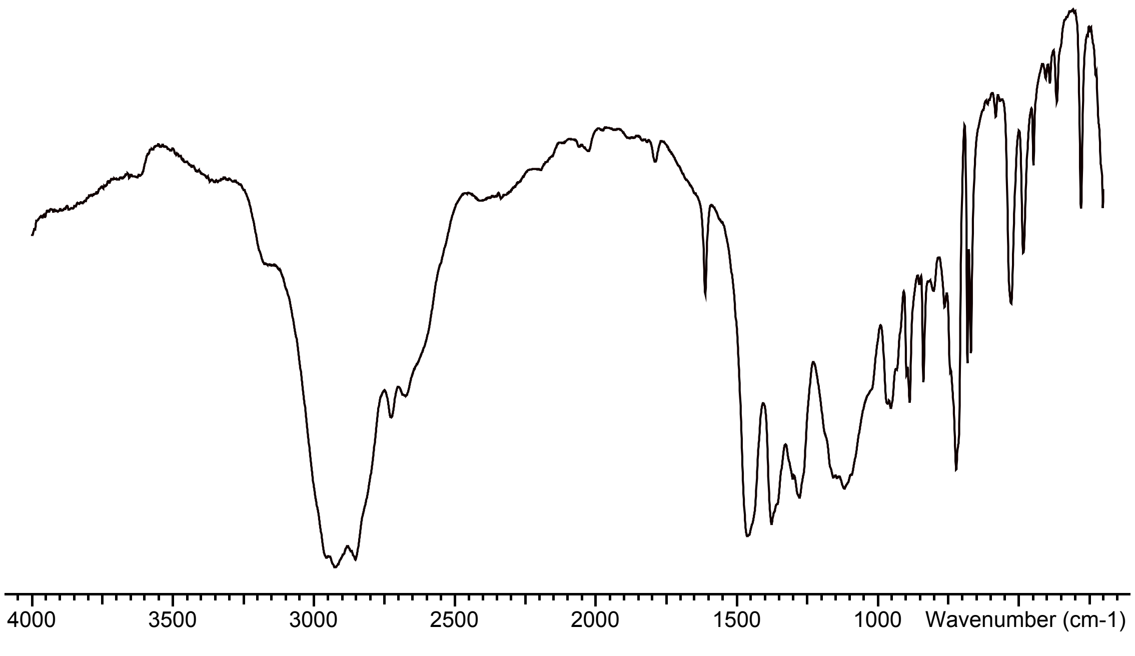

Figure S7 $[SiBr_3(PMe_3)_2][BAr^F]$

Figure S7(a) ^1H NMR spectrum of $[SiBr_3(PMe_3)_2][BAr^F]$ (CD_2Cl_2, 298 K), * = $[HPMe_3]^+$

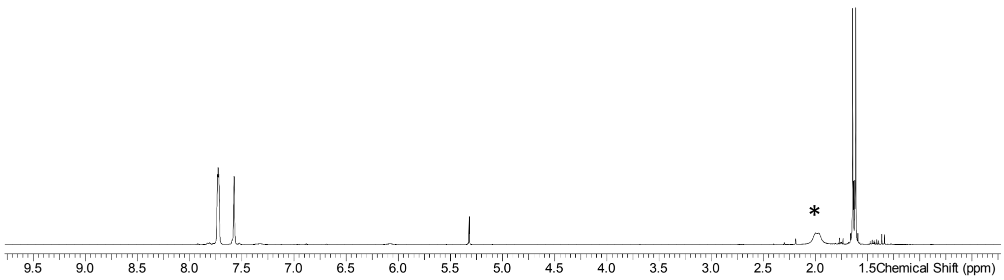

Figure S7(b) $^{31}P\{^1H\}$ NMR spectrum of $[SiBr_3(PMe_3)_2][BAr^F]$ (CD_2Cl_2, 298 K), * = $[HPMe_3]^+$

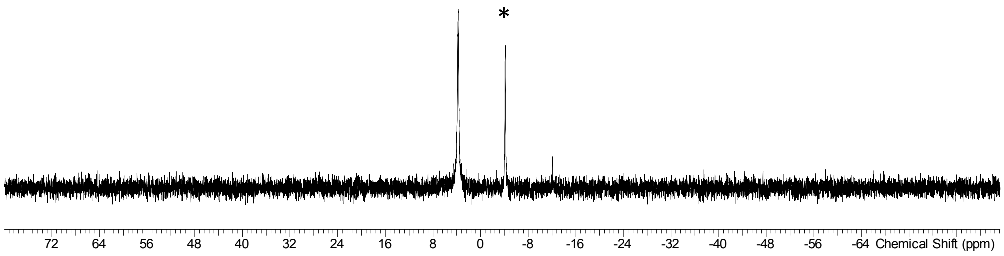

Figure S7(c) $^{31}P\{^1H\}$ NMR spectrum of $[SiBr_3(PMe_3)_2][BAr^F]$ (CD_2Cl_2, 183 K), * = $[HPMe_3]^+$

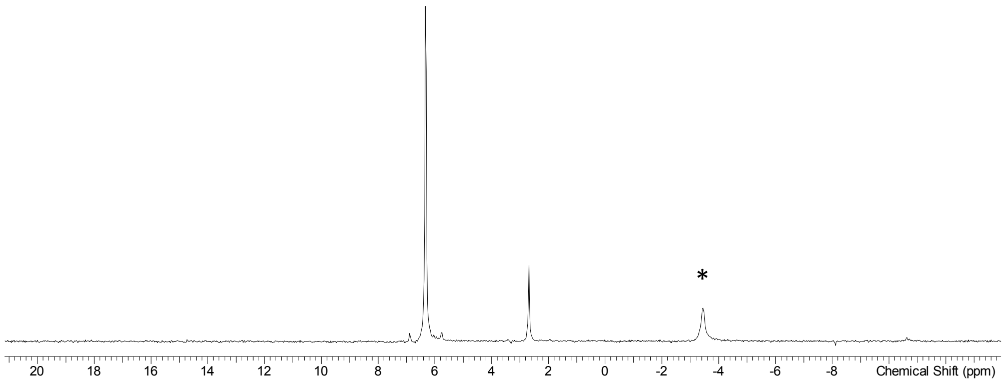

Figure 7(d) ^{29}Si NMR spectrum of $[SiBr_3(PMe_3)_2][BAr^F]$ (CD_2Cl_2, 183 K)

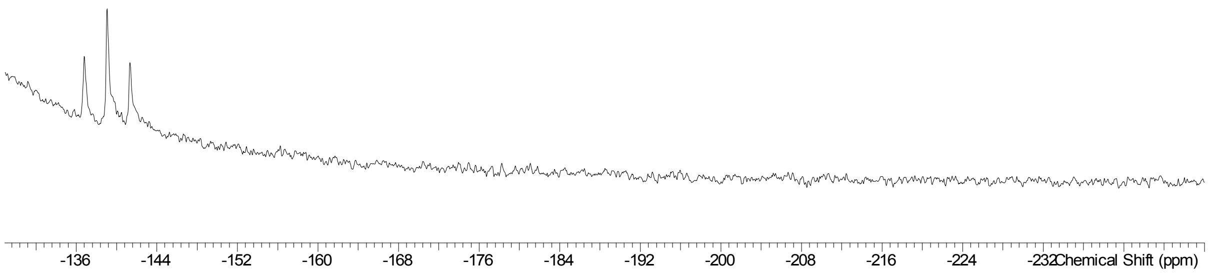

Figure 7(e) IR spectrum of $[SiBr_3(PMe_3)_2][BAr^F]$ (Nujol/cm^{-1})

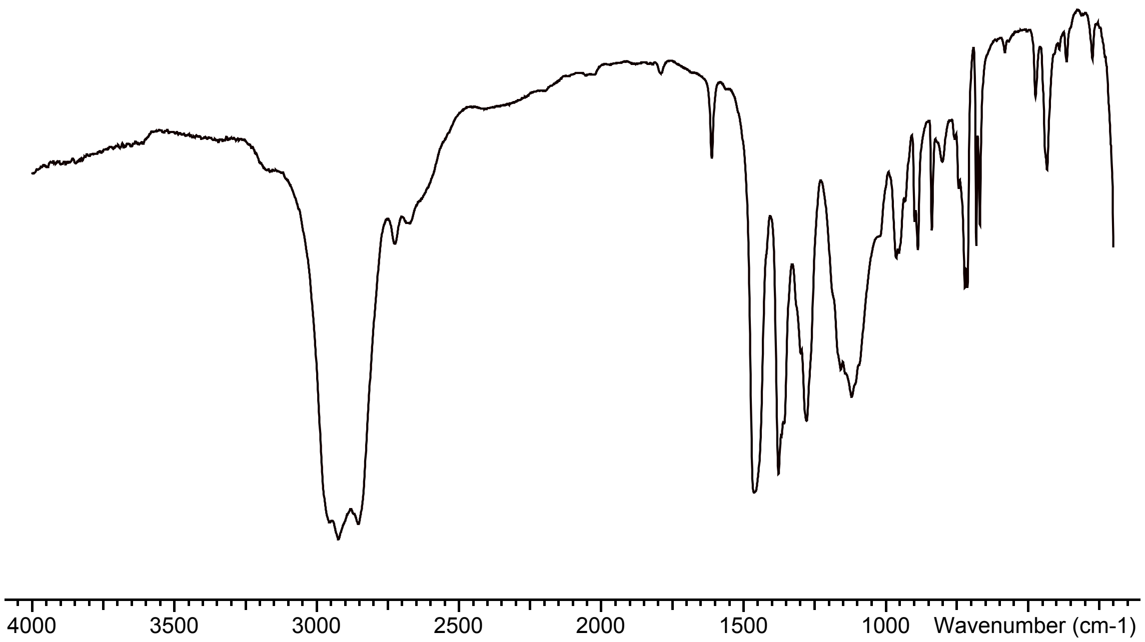

Figure S8 [$SiCl_3(PMe_3)_2(OTf)$]

Figure S8(a) ^1H NMR spectrum of [$SiCl_3(PMe_3)_2(OTf)$] (CD_2Cl_2, 298 K), * = [$HPMe_3$]$^+$

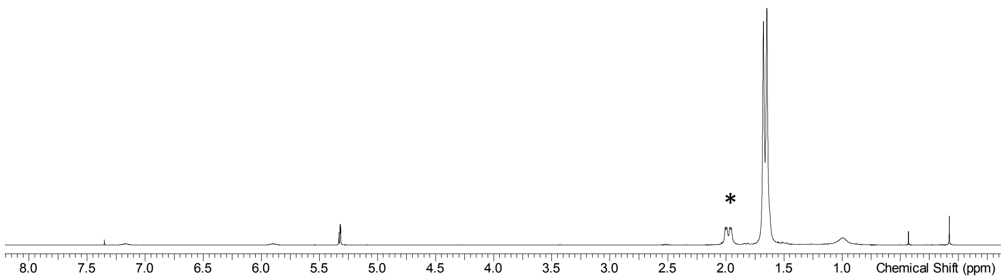

Figure S8(b) $^{19}F\{^1H\}$ NMR spectrum of [$SiCl_3(PMe_3)_2(OTf)$] (CD_2Cl_2, 298 K)

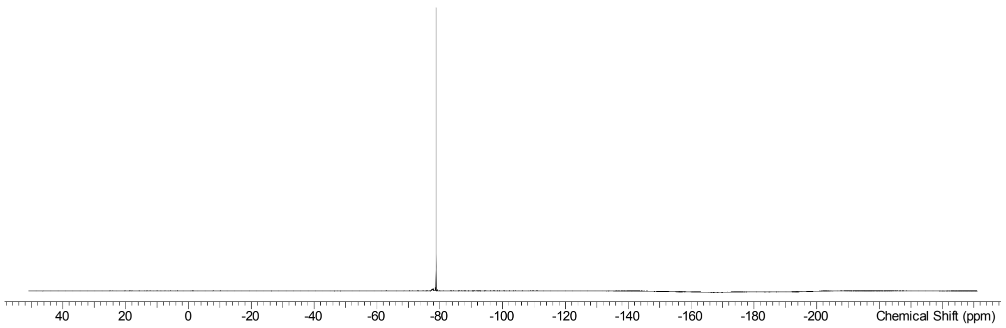

Figure S8(c) $^{31}P\{^1H\}$ NMR spectrum of [$SiCl_3(PMe_3)_2(OTf)$] (CD_2Cl_2, 298 K), * = [$HPMe_3$]$^+$

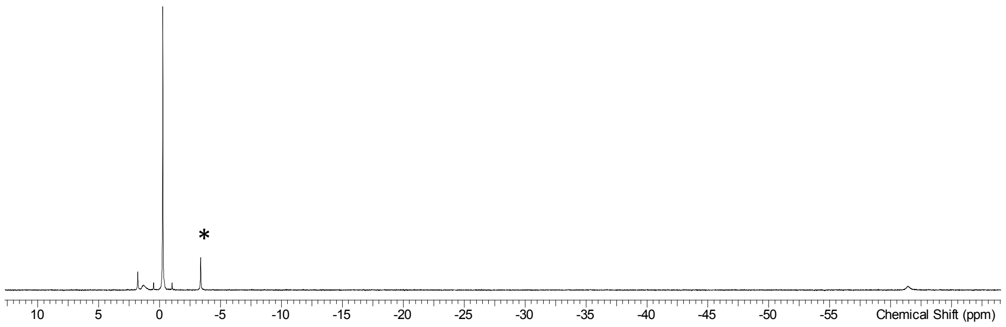

Figure S8(d) ^{29}Si NMR spectrum of $[SiCl_3(PMe_3)_2(OTf)]$ (CD_2Cl_2, 298 K)

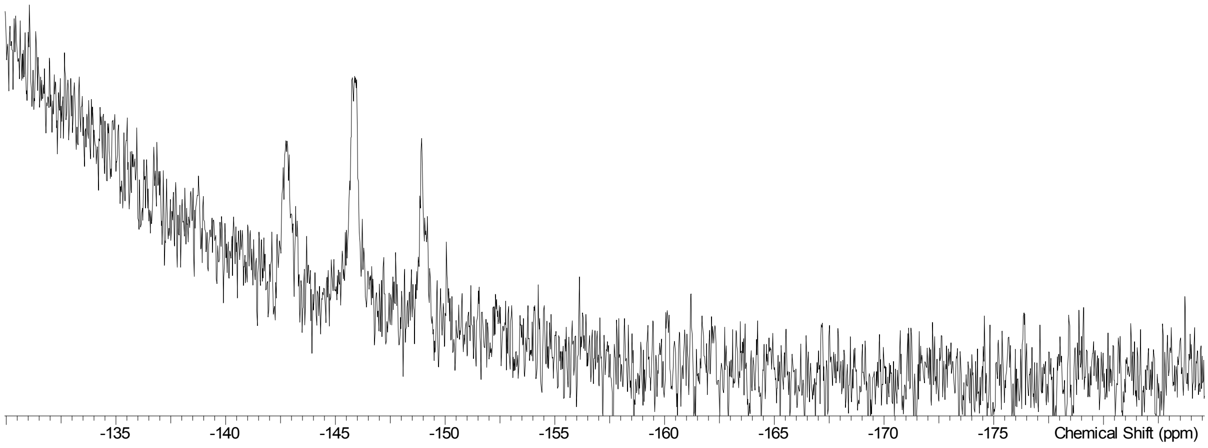

Figure S8(a) IR spectrum of $[SiCl_3(PMe_3)_2(OTf)]$ (Nujol/cm^{-1})

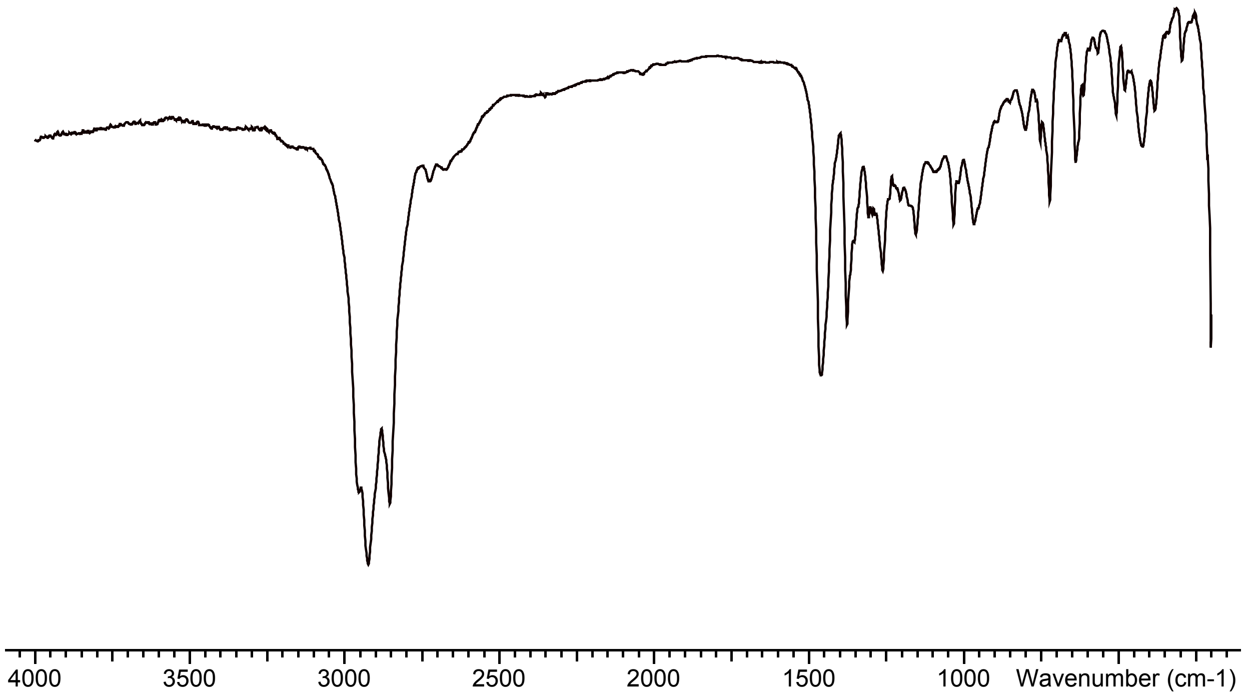

Figure S9 [$SiCl_2(PMe_3)_2(OTf)_2$]

Figure S9(a) ^{1}H NMR spectrum of [$SiCl_2(PMe_3)_2(OTf)_2$] (CD_2Cl_2, 298 K), * = [$HPMe_3$]$^+$; ^ = [$SiCl_3(PMe_3)_2(OTf)$]

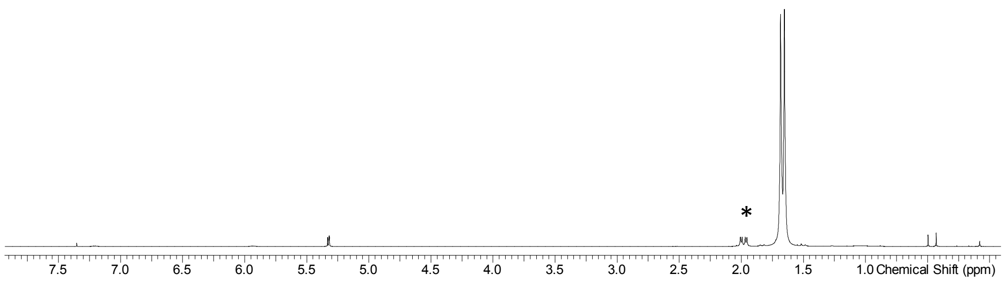

Figure S9(b) ^{19}F{^{1}H} NMR spectrum of [$SiCl_2(PMe_3)_2(OTf)_2$] (CD_2Cl_2, 298 K)

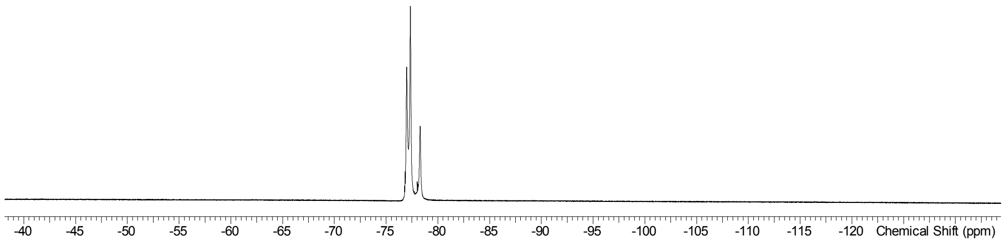

Figure S9(c) ^{31}P{^{1}H} NMR spectrum of [$SiCl_2(PMe_3)_2(OTf)_2$] (CD_2Cl_2, 298 K), * = [$HPMe_3$]$^+$; ^ = [$SiCl_3(PMe_3)_2(OTf)$]

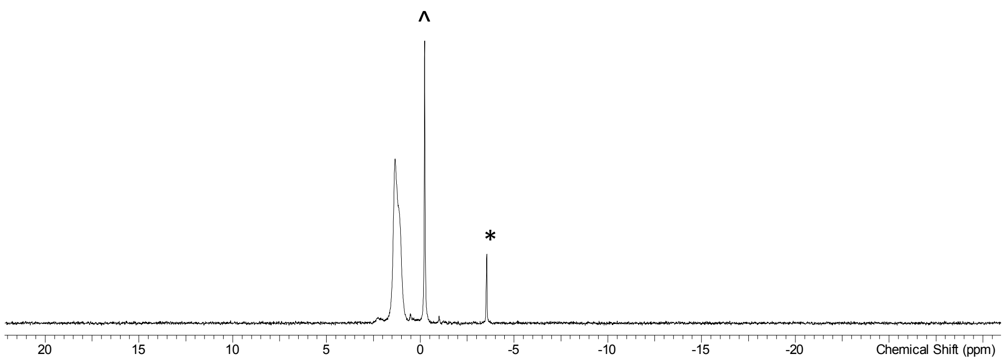

Figure S9(d) ^{29}Si NMR spectrum of $[SiCl_2(PMe_3)_2(OTf)_2]$ (CD_2Cl_2, 298 K)

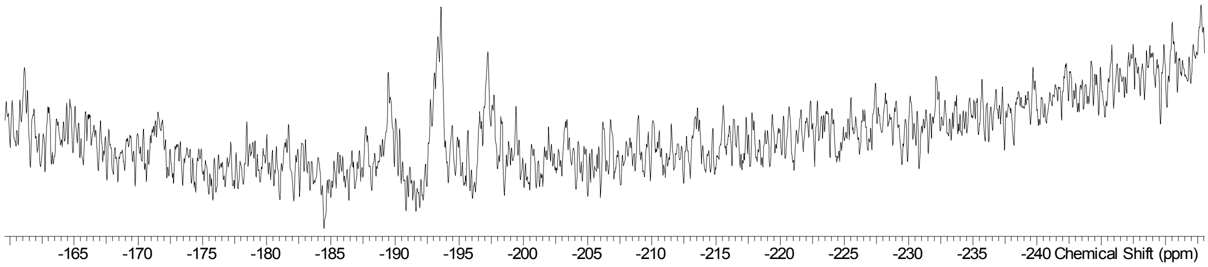

Figure S9(e) IR spectrum of $[SiCl_2(PMe_3)_2(OTf)_2]$ (Nujol/cm^{-1})

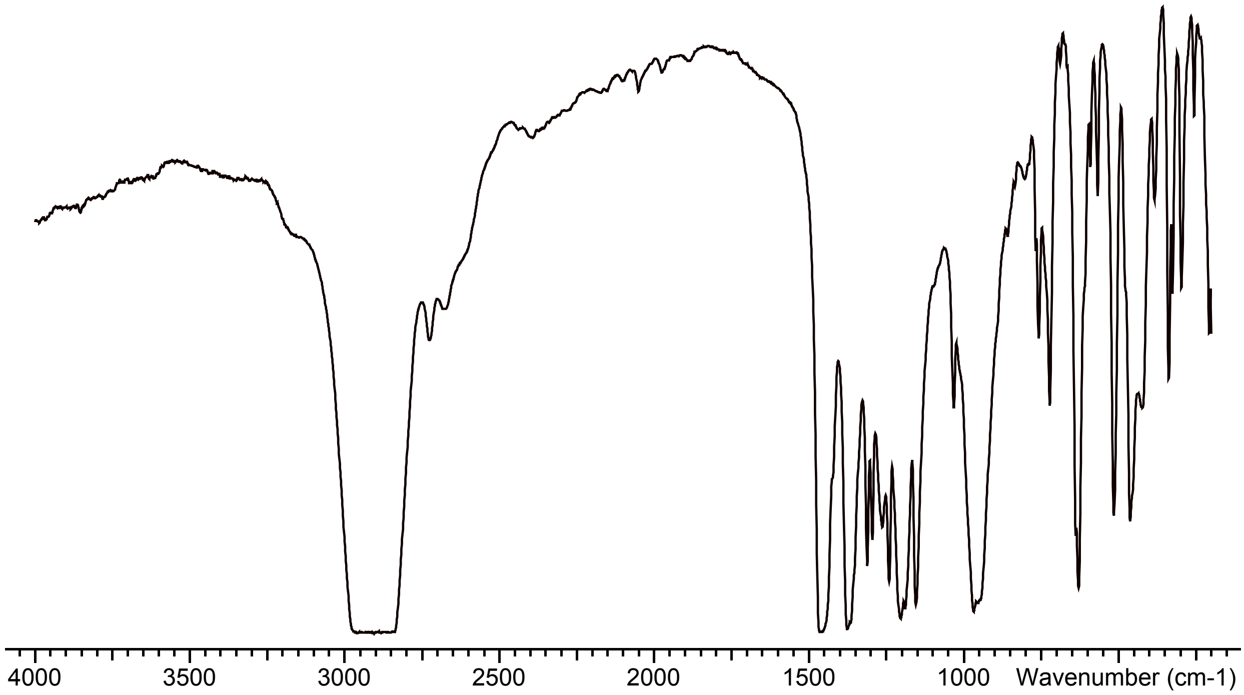

Figure S10 Frontier Orbital representations for [$SiF_4(PMe_3)_2$]

HOMO (-7.06 eV)	LUMO (0.864 eV)
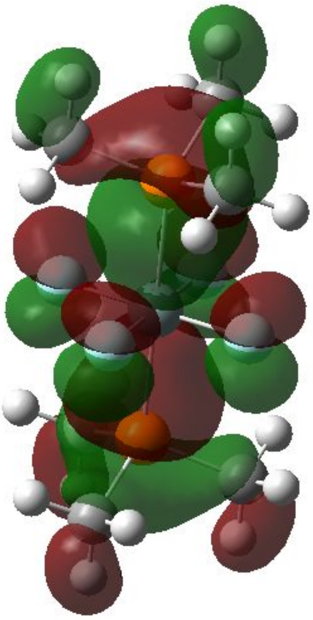	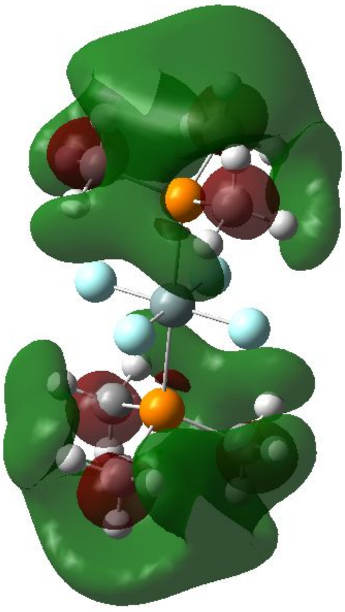
HOMO-1 (-7.58 eV)	LUMO+1 (0.930 eV)
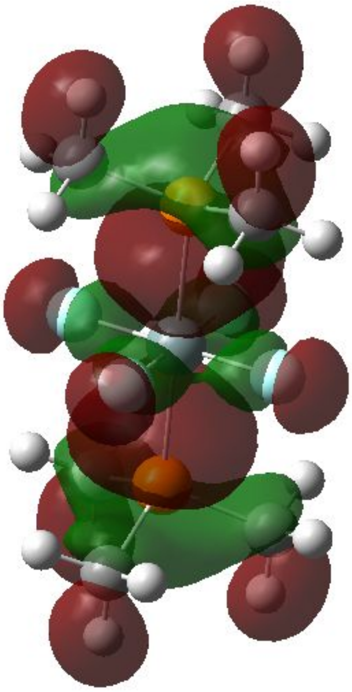	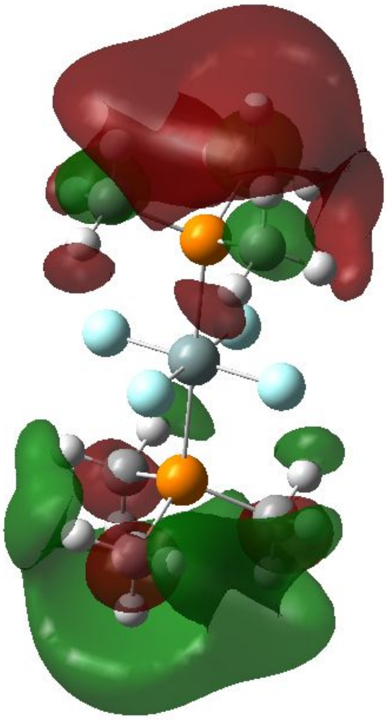

HOMO-2 (-8.30 eV)	LUMO+2 (1.32 eV)
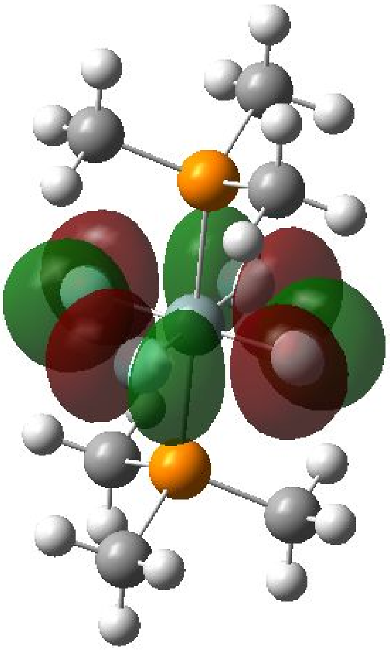	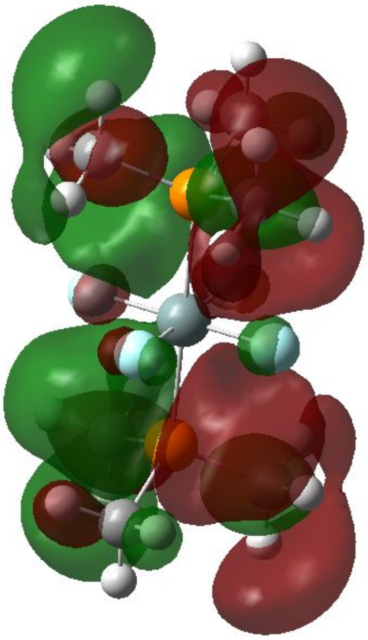

Figure S11 Frontier Orbital representations for $[SiF_3(PMe_3)_2]^+$

HOMO (-11.7 eV)	LUMO(-2.97 eV)
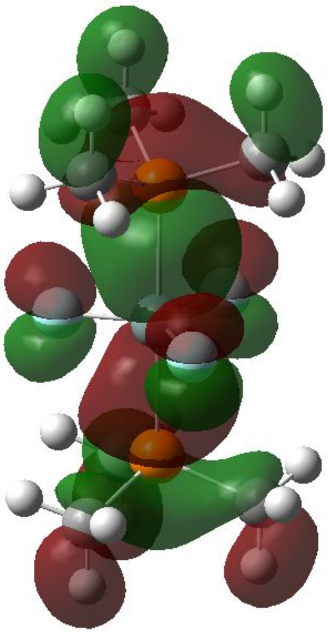	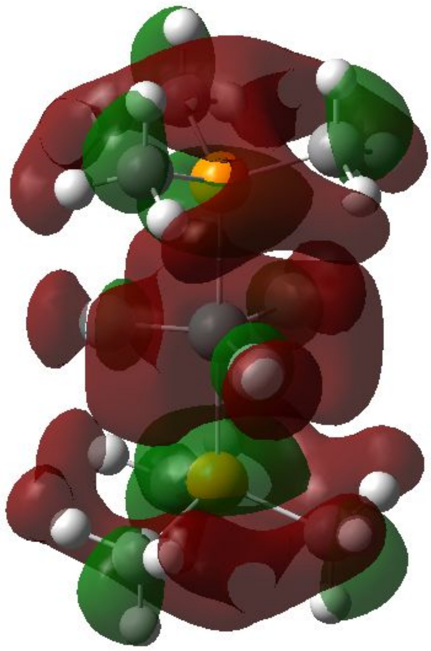

HOMO-1 (-12.0 eV)	LUMO+1 (-2.97 eV)
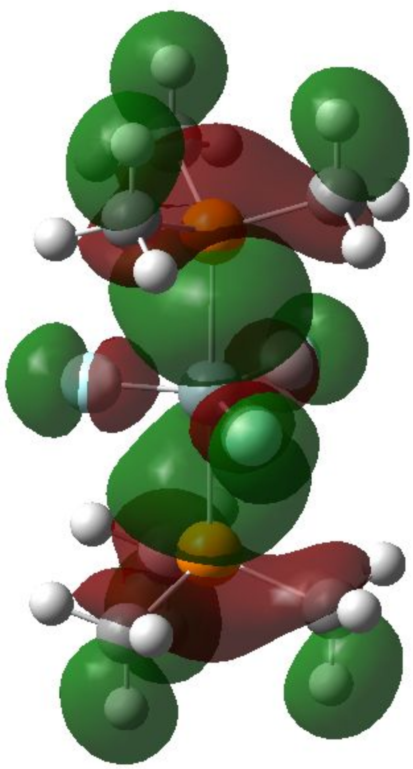	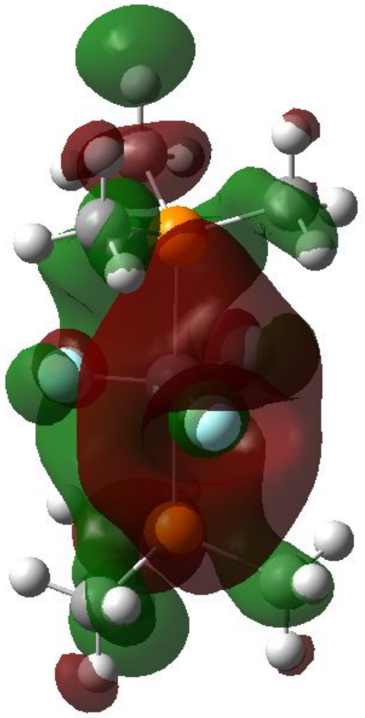
HOMO-2 (-13.6 eV)	LUMO+2 (-2.96 eV)
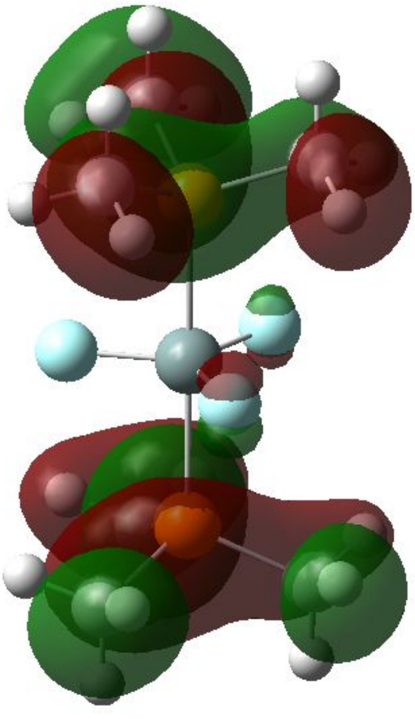	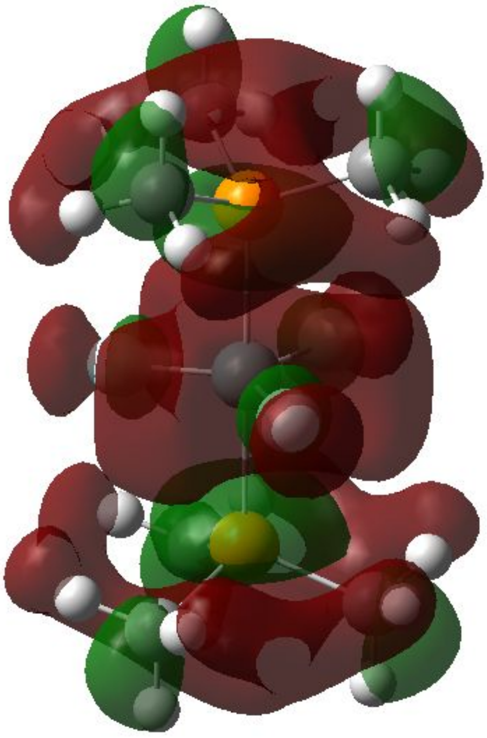

Figure S12 Frontier Orbital representations for [$SiCl_4(PMe_3)_2$]

HOMO (-6.90 eV)	LUMO (-1.35 eV)
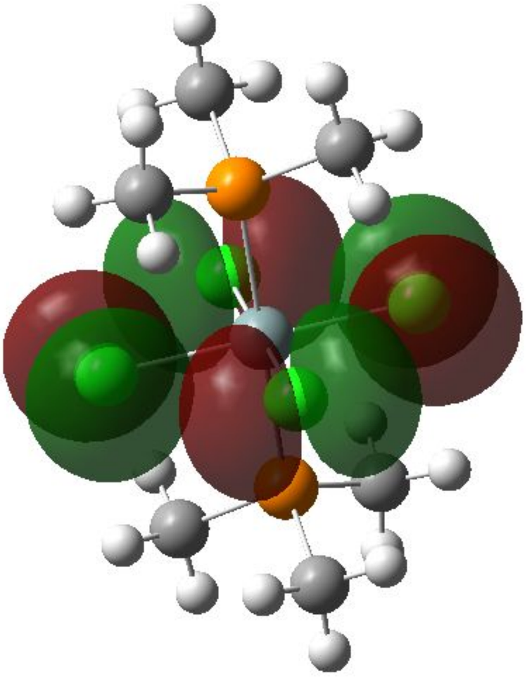	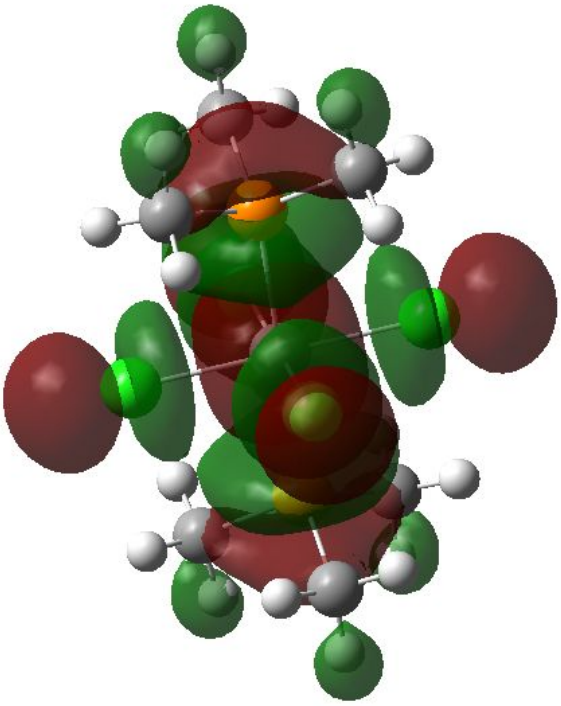
HOMO-1 (-7.40 eV)	LUMO+1 (0.629 eV)
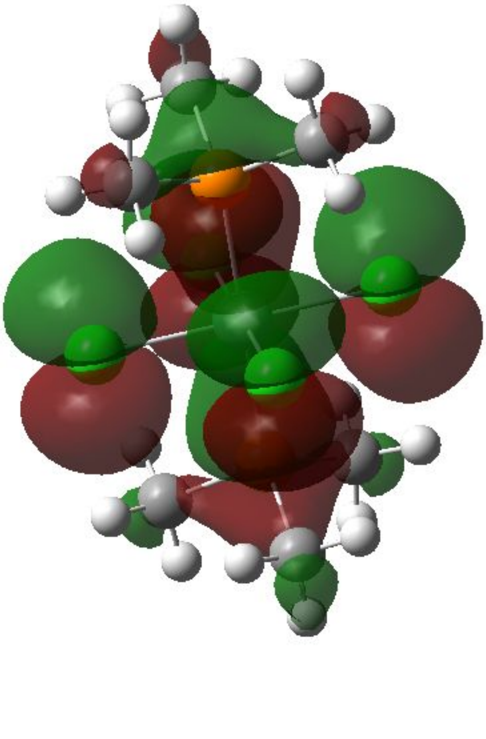	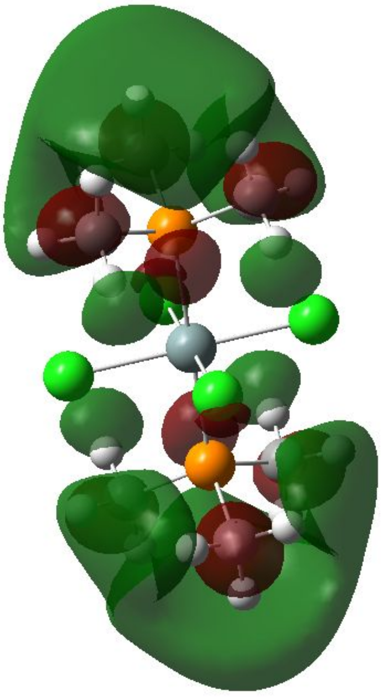

HOMO-2 (-7.57 eV)	LUMO+2 (0.660 eV)
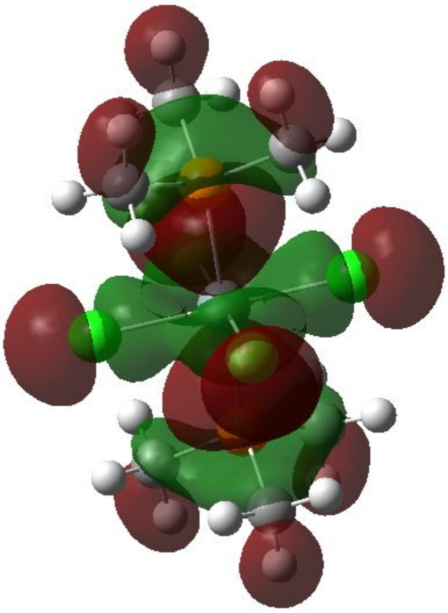	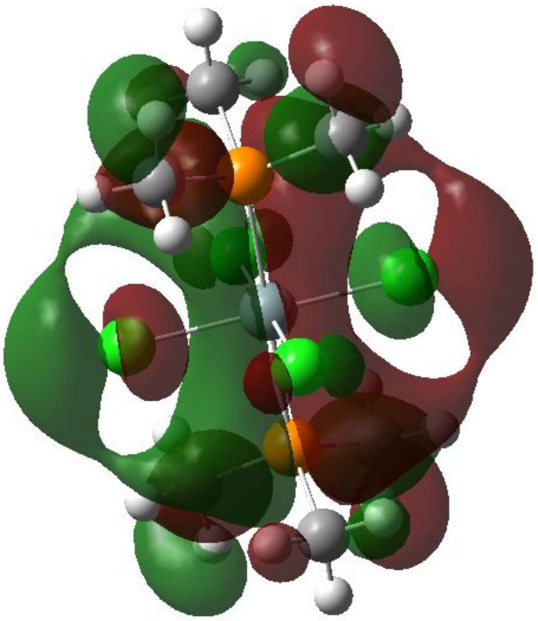

Figure S13 Frontier Orbital representations for [$SiCl_3(PMe_3)_2$]+

HOMO (-11.3 eV)	LUMO (-4.86 eV)
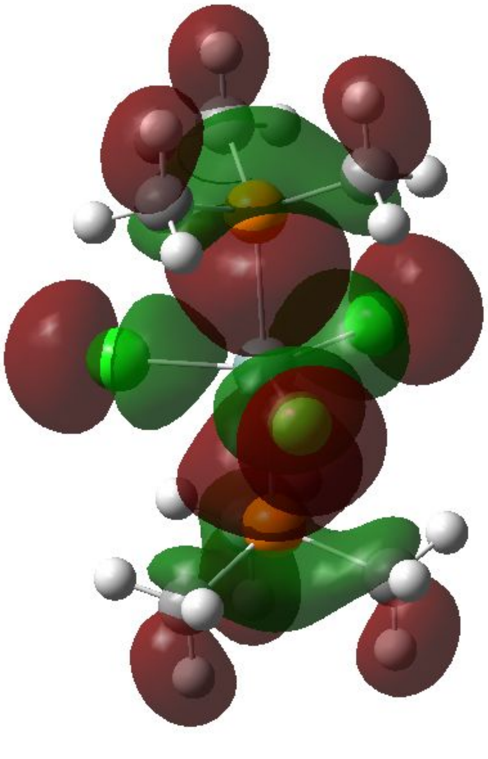	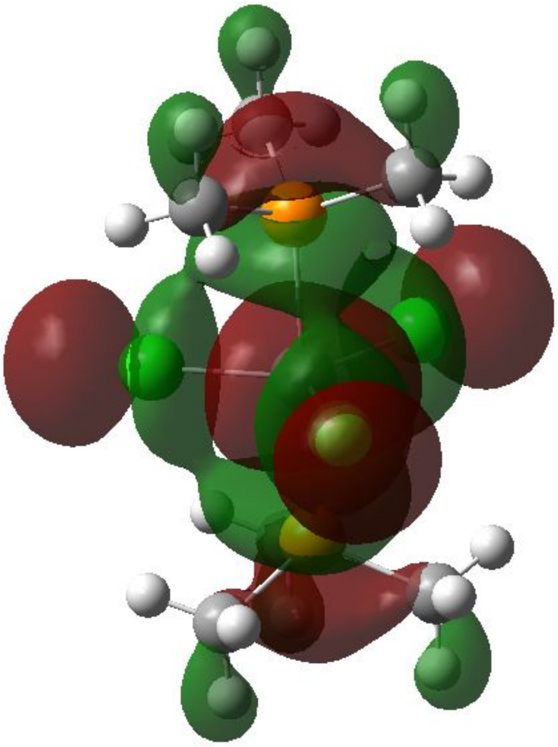

HOMO-1 (-11.4 eV)	LUMO+1 (-3.44 eV)
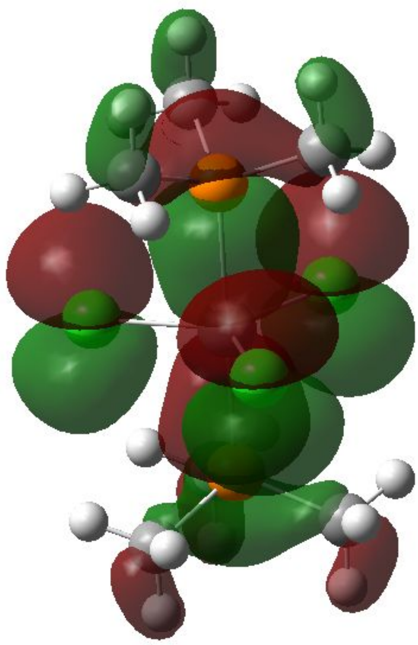	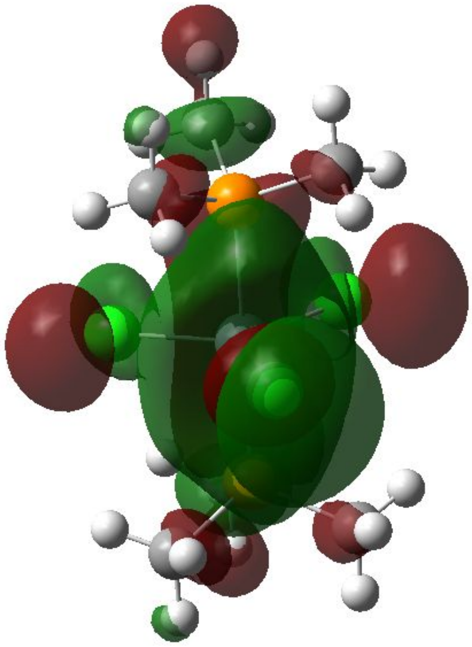
HOMO-2 (12.3 eV)	LUMO+2 (-3.44 eV)
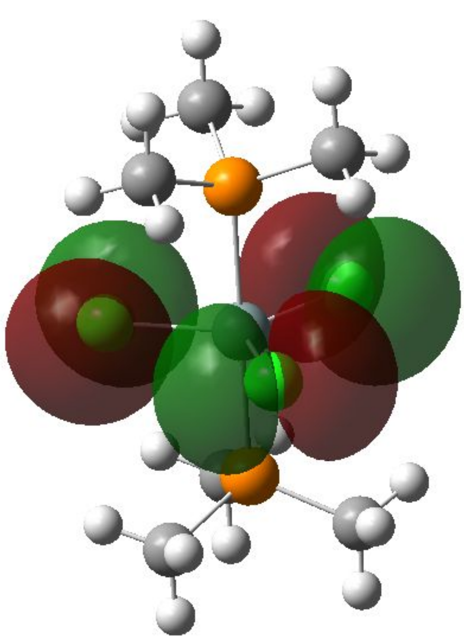	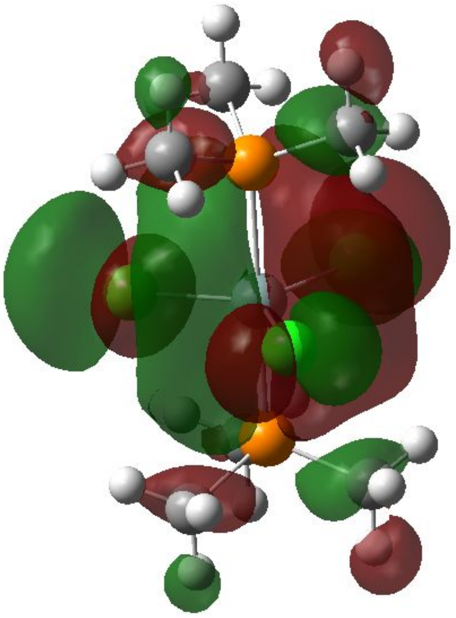

Figure S14 Frontier Orbital representations for [$SiBr_4(PMe_3)_2$]

HOMO (-6.28 eV)	LUMO (-0.245 eV)
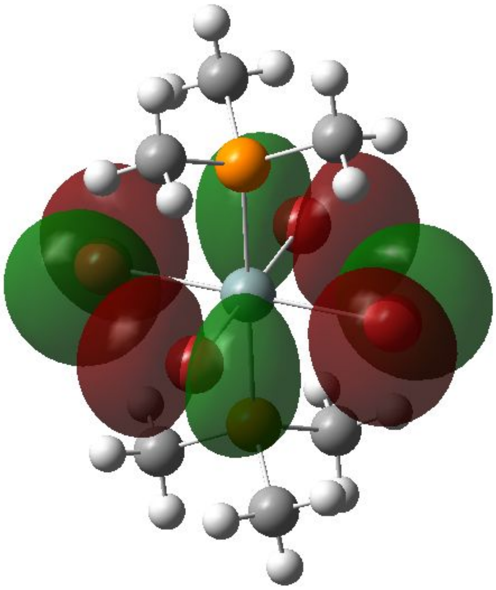	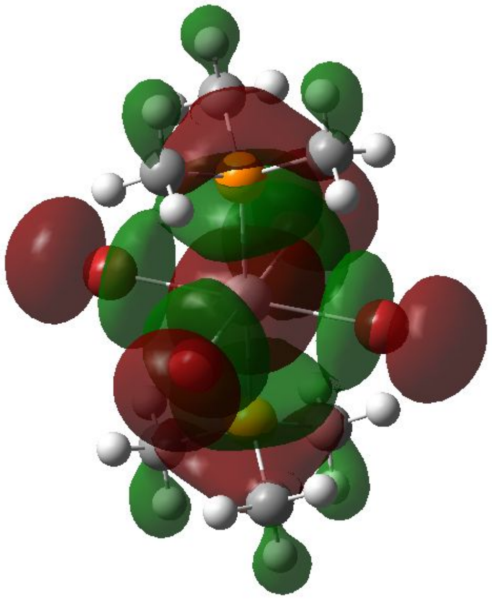
HOMO-1 (-6.92 eV)	LUMO+1 (0.127 eV)
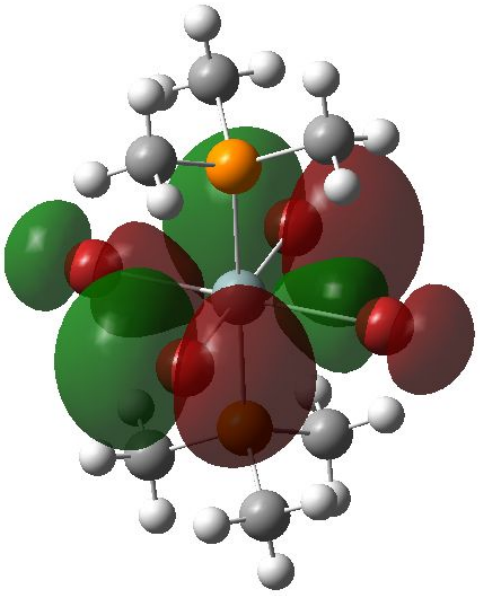	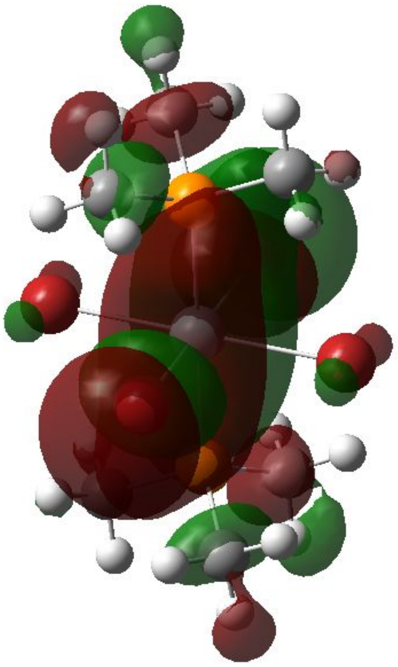

HOMO-2 (-6.94 eV)	LUMO+2 (0.140 eV)
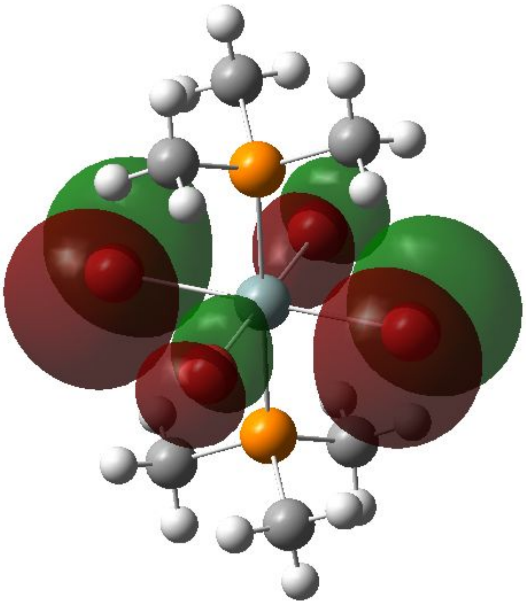	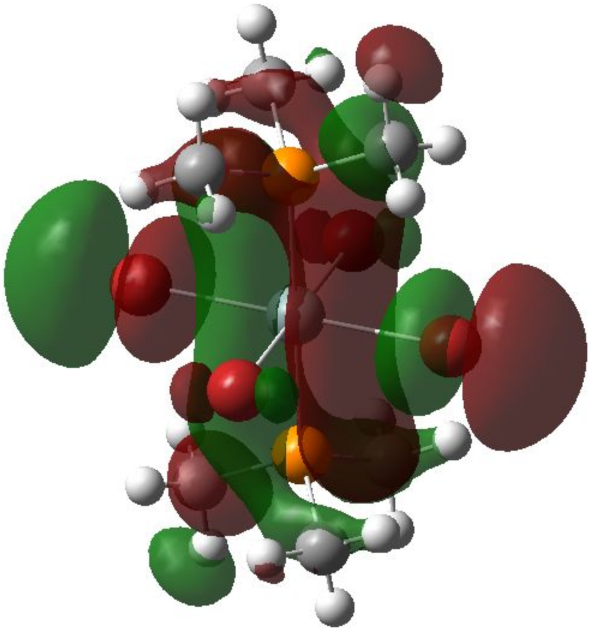

Figure S15 Frontier Orbital representations for $[SiBr_3(PMe_3)_2]^+$

HOMO (-11.1 eV)	LUMO (-5.67 eV)
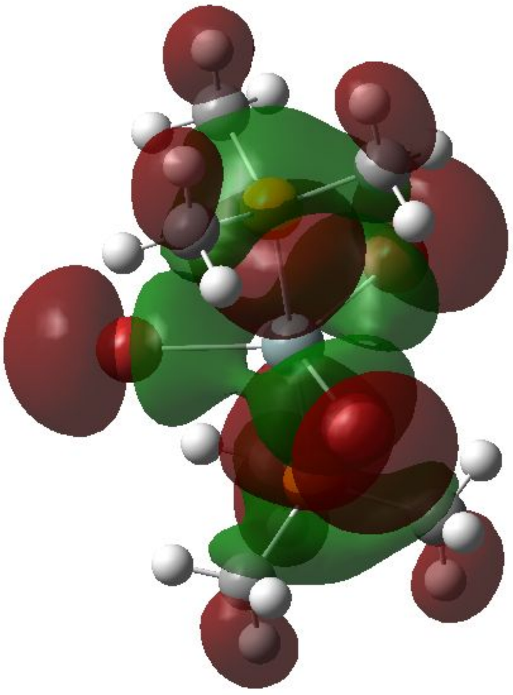	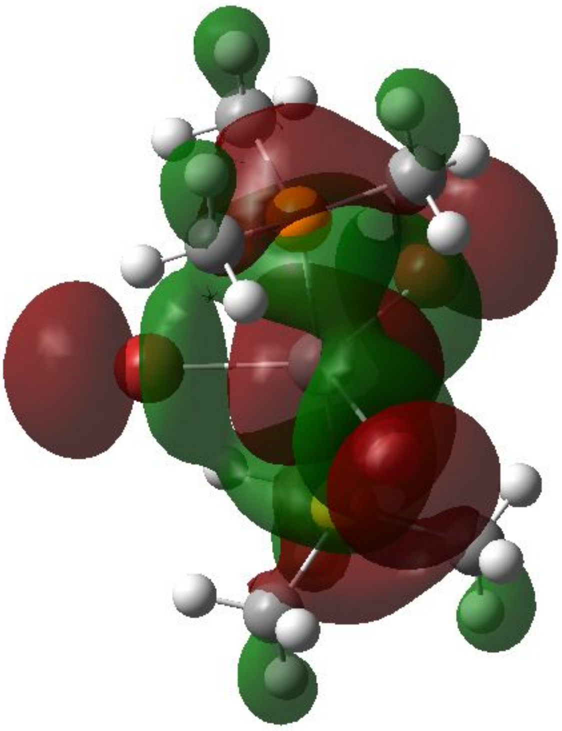

HOMO-1 (-11.1 eV)	LUMO+1 (-4.01 eV)
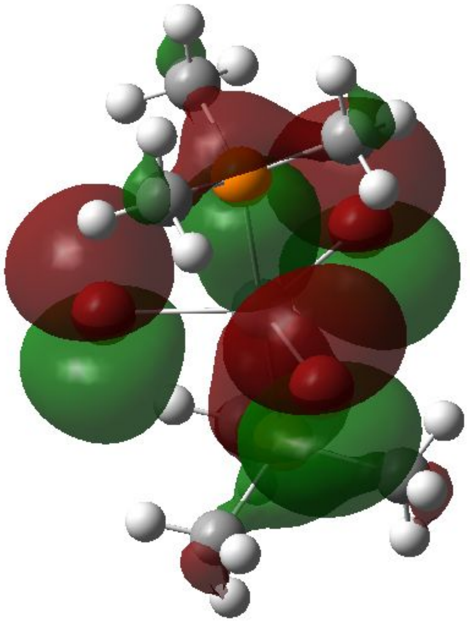	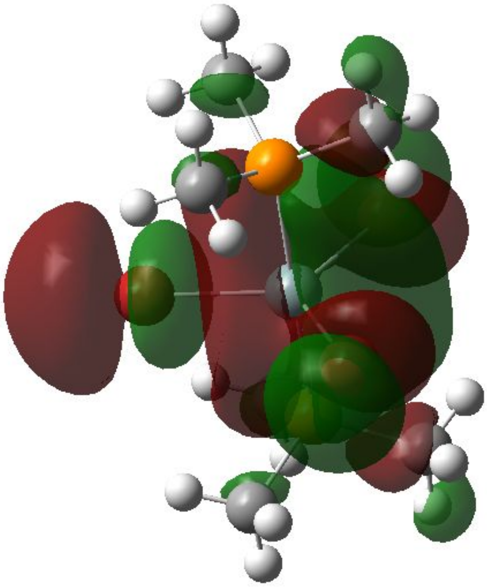
HOMO-2 (-11.4 eV)	LUMO+2 (-4.01 eV)
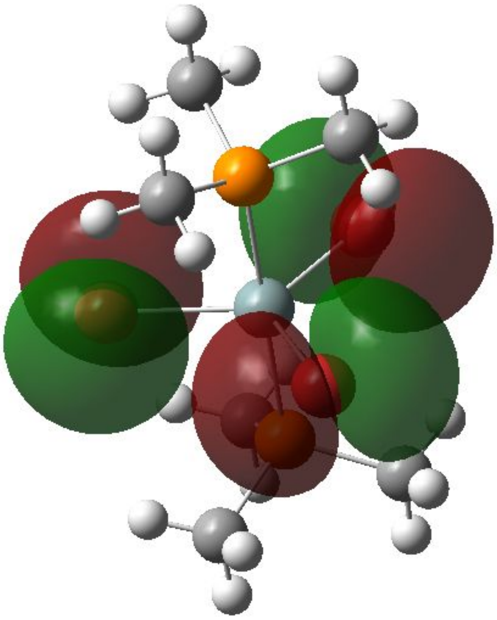	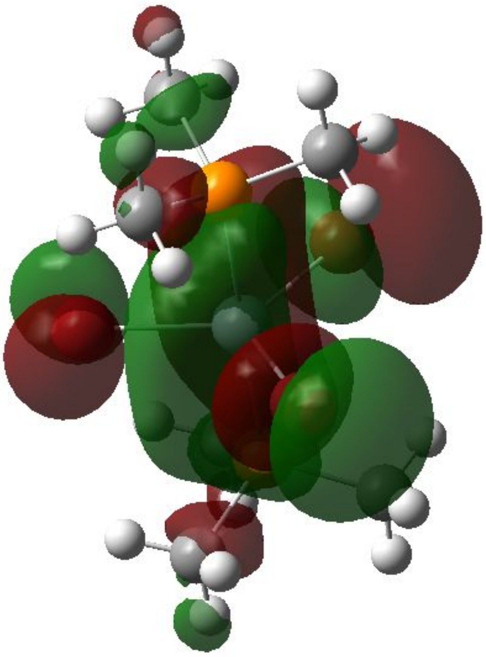

Figure S16 Frontier Orbital representations for [$SiI_4(PMe_3)_2$]

HOMO (-5.78 eV)	LUMO (-3.00 eV)
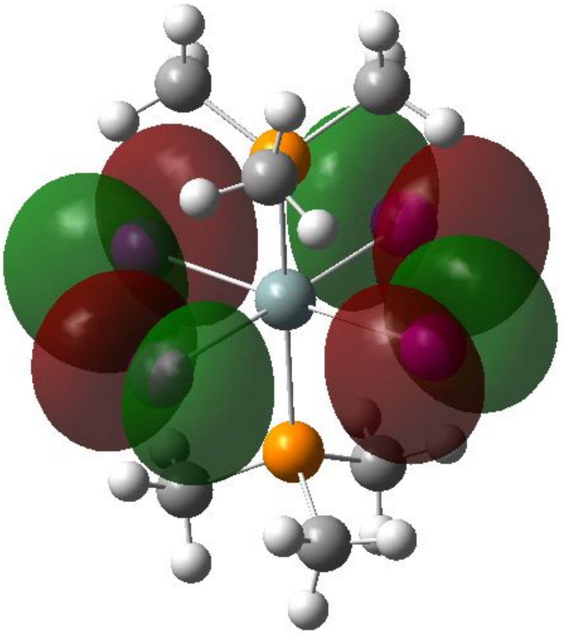	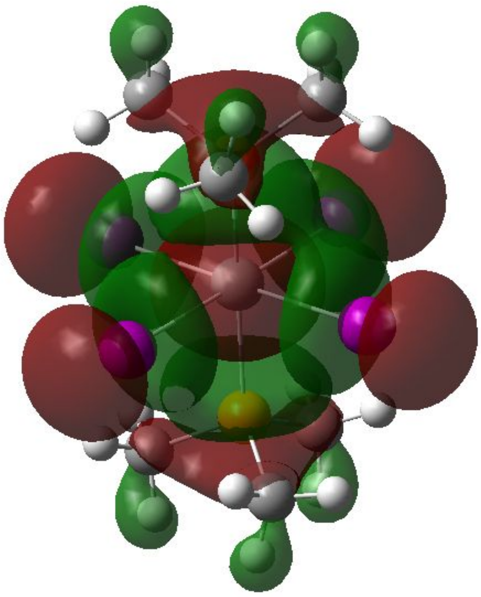
HOMO-1 (-6.42 eV)	LUMO+1 (-0.613 eV)
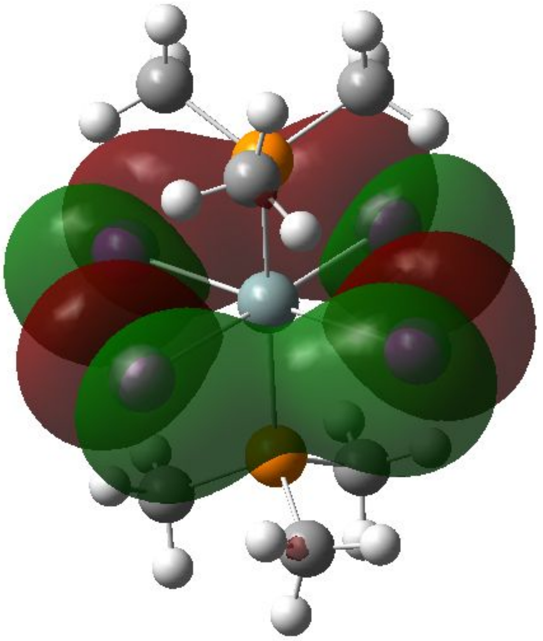	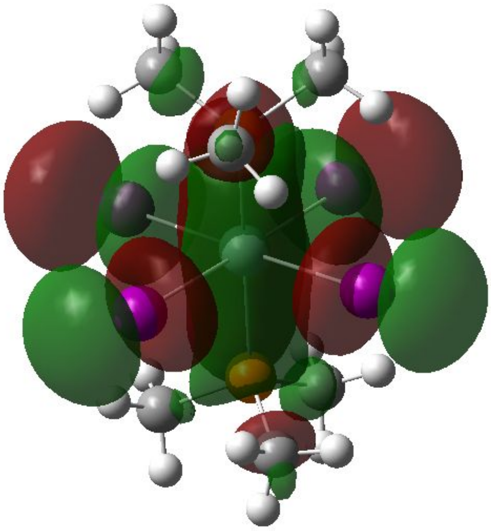

HOMO-2 (-6.47 eV)	LUMO+2 (-0.565 eV)
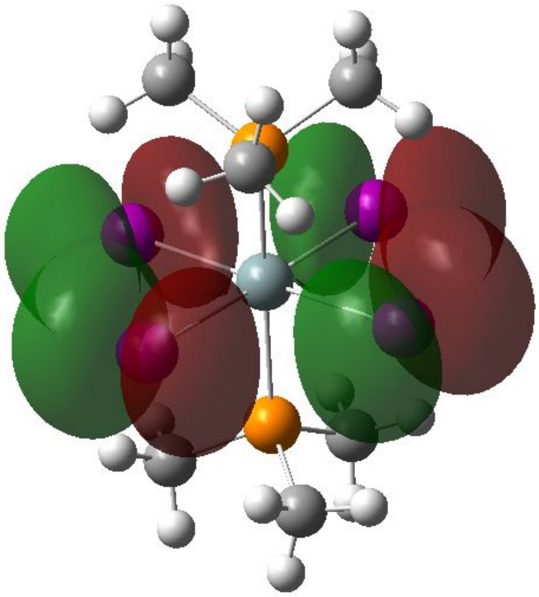	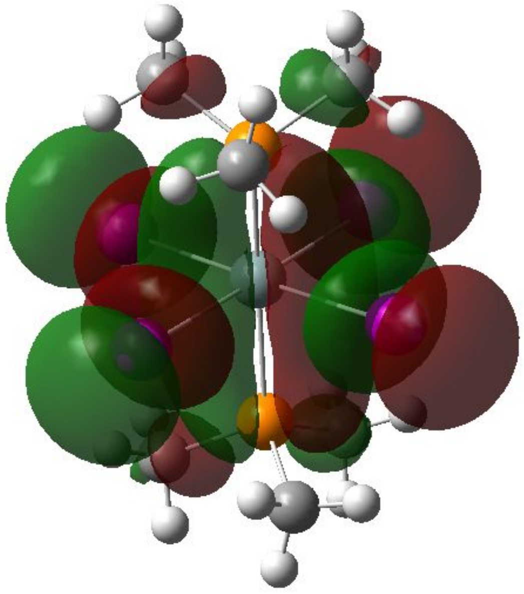

Figure S17 Frontier Orbital representations for $[SiI_3(PMe_3)_2]^+$

HOMO (-10.5 eV)	LUMO (-6.30 eV)
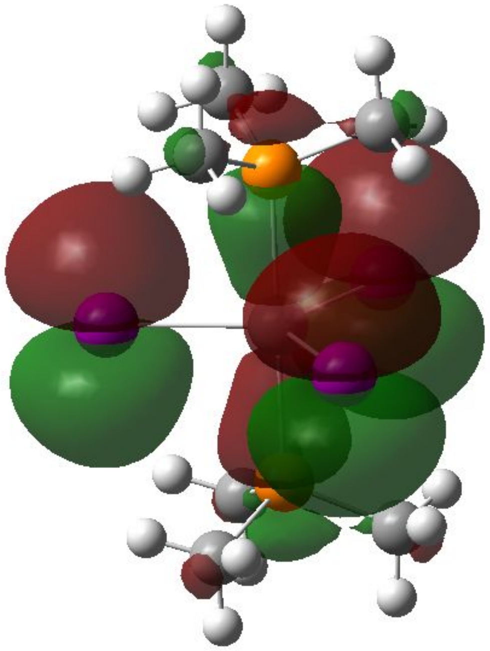	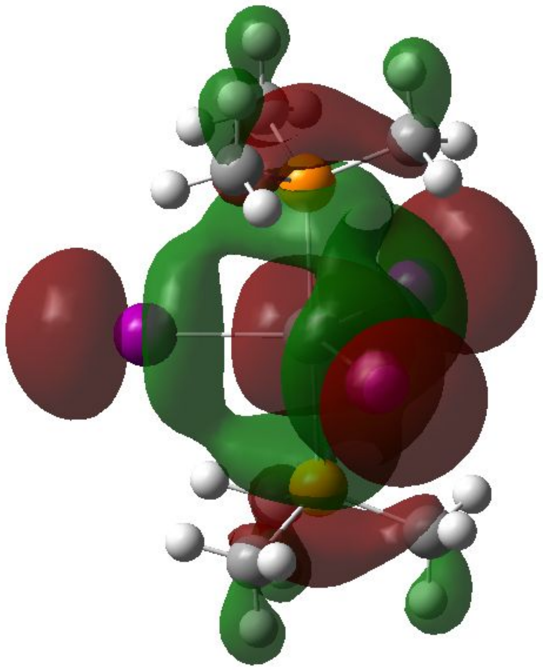

HOMO-1 (-10.5 eV)	LUMO+1 (-4.34 eV)
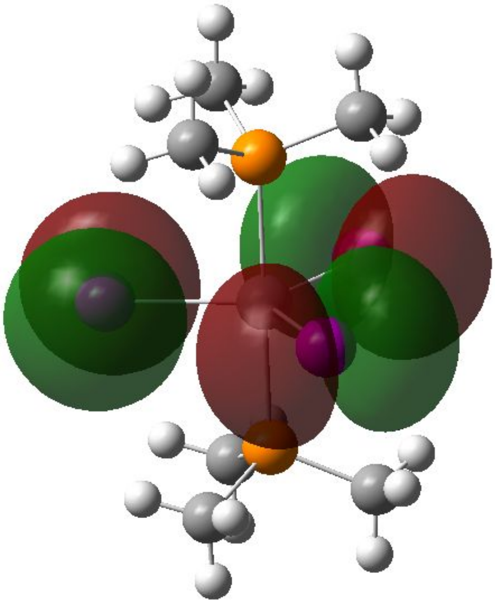	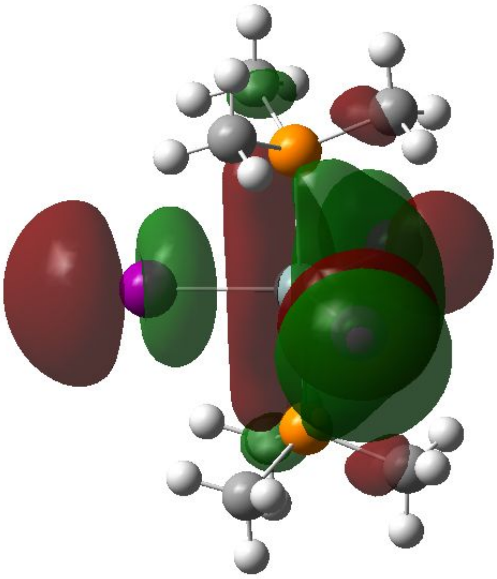
HOMO-2 (-10.6 eV)	LUMO+2 (-4.33 eV)
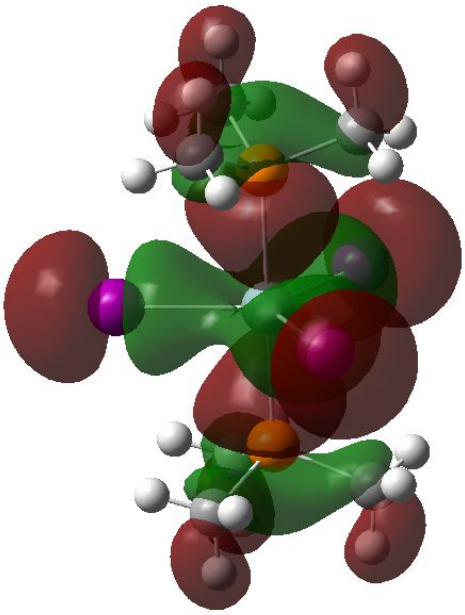	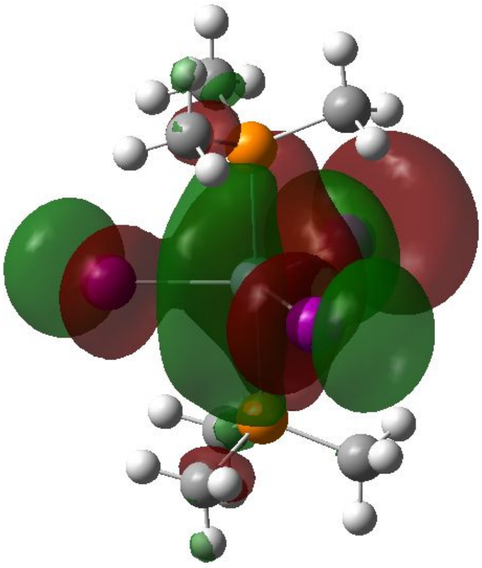
